# SKLB-677, an FLT3 and Wnt/β-catenin signaling inhibitor, displays potent activity in models of FLT3-driven AML

**DOI:** 10.1038/srep15646

**Published:** 2015-10-26

**Authors:** Shuang Ma, Ling-Ling Yang, Ting Niu, Chuan Cheng, Lei Zhong, Ming-Wu Zheng, Yu Xiong, Lin-Li Li, Rong Xiang, Li-Juan Chen, Qiao Zhou, Yu-Quan Wei, Sheng-Yong Yang

**Affiliations:** 1 State Key Laboratory of Biotherapy and Cancer Center, West China Hospital, Sichuan University/Collaborative Innovation Center of Biotherapy, Chengdu, 610041, China; 2 Department of Hematology & Research Laboratory of Hematology, West China Hospital, Sichuan University, Chengdu, 610041, China; 3 Department of Clinical Medicine, School of Medicine, Nankai University, Tianjin, 300071, China

## Abstract

FLT3 has been identified as a valid target for the treatment of acute myeloid leukemia (AML), and some FLT3 inhibitors have shown very good efficacy in treating AML in clinical trials. Nevertheless, recent studies indicated that relapse and drug resistance are still difficult to avoid, and leukemia stem cells (LSCs) are considered one of the most important contributors. Here, we report the characterization of SKLB-677, a new FLT3 inhibitor developed by us recently. SKLB-677 exhibits low nanomolar potency in biochemical and cellular assays. It is efficacious in animal models at doses as low as 1mg/kg when administrated orally once daily. In particular, SKLB-677 but not first-generation and second-generation FLT3 inhibitors in clinical trials has the ability to inhibit Wnt/β-catenin signaling; Wnt/β-catenin signaling is required for the development of LSCs, but not necessary for the development of adult hematopoietic stem cells (HSCs). This compound indeed showed considerable suppression effects on leukemia stem-like cells in *in vitro* functional assays, but had no influence on normal HSCs. Collectively, SKLB-677 is an interesting lead compound for the treatment of AML, and deserves further investigations.

Acute myeloid leukemia (AML) is a fast-growing and aggressive malignancy of the blood and bone marrow^1^,^2^. The elucidation of the molecular and biological underpinnings of AML has revealed that mutations and/or the aberrant expression of specific protein tyrosine kinases (PTKs) are important factors responsible for the occurrence and development of AML^3^. Among these PTKs, the FMS-like tyrosine kinase 3 (FLT3) is of particular importance. Activating mutations in FLT3 kinase are found in up to one-third of AML cases^4^,^5^. The most prevalent activating mutations are internal tandem duplications (ITD) in the juxtamembrane domain, which lead to constitutive, ligand-independent activation of the kinase^6^,^7^. Numerous studies have demonstrated that the FLT3-ITD mutation represents a driver mutation for the initiation and development of AML and is associated with a poor prognosis for overall survival^8^,^9^,^10^. FLT3 has thus been identified as a valid therapeutic target for AML treatment.

The recognition of the importance of FLT3-ITD and the FLT3 pathway in the initiation and development of AML has stimulated efforts to develop therapeutic inhibitors of FLT3^11^,^12^,^13^. A number of small-molecule tyrosine kinase inhibitors of FLT3 have been developed, including first-generation FLT3 inhibitors (which are multikinase inhibitors) such as CEP-701, MLN-518, BAY-43-9006 (sorafenib), and SU-11248 (sunitinib), and second-generation ones (which are selective FLT3 inhibitors) such as AC220^14^,^15^,^16^,^17^,^18^. Although some of these inhibitors have shown promising anti-leukemic activity in clinical trials, relapse or drug resistance often occurs^19^,^20^,^21^. The causes of relapse and drug resistance are complex, but leukemia stem cells (LSCs) are likely one of the most important contributors^22^,^23^,^24^. Recently, Wang and Armstrong^25^ demonstrated that the Wnt/β-catenin pathway is required for the development of LSCs in AML. In the same study, they also showed that Wnt/β-catenin signaling is not essential for the development of adult hematopoietic stem cells (HSCs)^25^. All of these results suggest that targeting the Wnt/β-catenin pathway may represent a new therapeutic strategy to eliminate LSCs and prevent AML relapse and drug resistance^25^,^26^. We hypothesized that agents that are able to simultaneously target FLT3 and the Wnt/β-catenin pathway might bring an improved clinical outcome in the treatment of AML. Therefore, we conducted designing and screening studies to identify agents that target both FLT3 and the Wnt/β-catenin pathway, which led to the discovery of 1-(4-(1H-pyrazolo[3,4-d]pyrimidin-4-yloxy)-3-fluorophenyl)-3-(5-tert-butylisoxazol-3-yl)urea, termed SKLB-677 (Fig. 1a). This compound exhibits sub-nanomolar binding affinity for FLT3 and good activity in blocking the Wnt/β-catenin signaling pathway. It displays potent anti-cancer activity in models of FLT3-driven AML and considerable inhibitory ability to leukemia stem-like cells or leukemia-initiating cells (LICs) in Hoechst side population (SP) assays and long-term culture initiating cell (LTC-IC) assays. Taking together, SKLB-677 is a promising new lead compound for the treatment of AML.

## Results

### Discovery of SKLB-677

**Figure 1:**
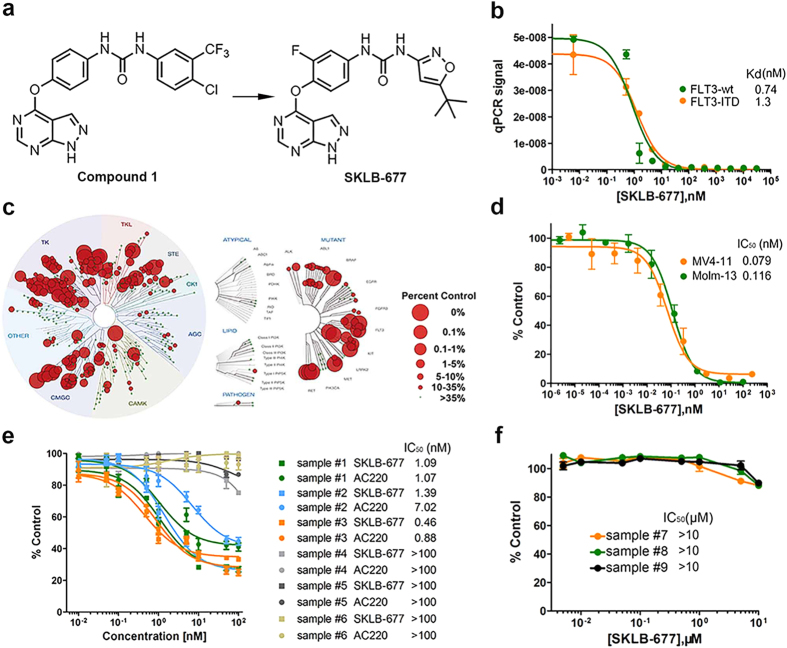
The chemical structure of SKLB-677 and its *in vitro* bioactivities. (**a**) Chemical structures of compound **1** and SKLB-677. (**b**) Binding affinities of SKLB-677 against human FLT3-wt and the FLT3-ITD mutant measured by KINOMEscan kinase binding assays (Ambit Biosciences). The y-axis indicates the concentrations of the tested kinases, which are represented by the signal of qPCR (the tested kinases were tagged with DNA and detected using qPCR assays). All data points are means of triplicates ± SD. IC_50_ values are presented. (**c**) SKLB-677 was screened against a KINOMEscan ( http://www.kinomescan.com) panel of 456 kinase assays. The red circles indicate bound kinases, and the circle size indicates the degree of binding affinity. The complete dataset is shown in Supplementary Table S3. (**d**) MV4-11 or Molm-13 cells were incubated in the presence of various concentrations of SKLB-677. The viabilities of the cultured cells after 72 h were measured by MTT assays. All data points are means of triplicates ± SD. IC_50_ values are presented. (**e**), Primary AML PBSCs were incubated in the presence of various concentrations of SKLB-677 and/or AC220. The viabilities of the cultured cells after 72 h were measured by the CellTiterGlo assays. The primary AML cells were obtained from 6 patients (sample #1, #2, #3: FLT3-ITD positive, sample #4, #5, #6: FLT3-ITD negative). All data points are means of triplicates ± SEM. IC_50_ values are presented in the right-hand column. (**f**) Normal PBMCs were incubated in the presence of various concentrations of SKLB-677 for 72 h, followed by the measurement using CellTiterGlo assays. All data points are means of triplicates ± SEM. All the IC_50_ values are >10 μM.

SKLB-677 was derived from a previously identified FLT3 inhibitor^27^, 1-(4-(1*H*-pyrazolo[3,4-*d*]pyrimidin- 4-yloxy)phenyl)- 3-(4-chloro-3-(trifluoro methyl)phenyl)urea (**1**, Fig. 1a), which displayed moderate inhibitory potency against FLT3 with an IC_50_ value of 39 nM, and very weak activity in inhibiting Wnt/β-catenin signaling. A series of structural modifications were conducted on compound **1**, which mainly include: (1) substitution at the 3-position of the urea moiety with various 5-membered heteroaromatic rings; (2) substitution at the *N*-1 position of the pyrazolo[3,4-*d*]pyrimidine with different groups; (3) substitution at the 2-position of the 1-phenyl ring of the urea moiety with various substituents. Structure-activity relationship (SAR) studies yielded several new compounds with significantly improved potency against FLT3 and potent activity in inhibiting Wnt/β-catenin signaling. SKLB-677 is one of the best compounds, which not only showed higher potencies in inhibiting FLT3 activation and Wnt/β-catenin signaling, but also had better pharmacokinetic properties. A brief description of the chemical characterization of SKLB-677 as well as a comparison between SKLB-677 and compound **1** is provided in Supplementary Data (Supplementary Chemistry, Fig. S1, Table S1 and S2).

### Potency of FLT3 inhibition and kinase selectivity of SKLB-677

The binding affinities of SKLB-677 with FLT3 (wild-type, wt) and its mutants FLT3-D835H, FLT3-D835Y, FLT3-ITD, and other selected kinases were measured by KINOMEscan kinase binding assays (Ambit Biosciences). The results are presented in Table 1. SKLB-677 has sub-nanomolar or low nanomolar binding affinity for FLT3-wt (0.74 nM, Fig. 1b), FLT3-D835H (0.74 nM), FLT3-D835Y (1.3 nM), and FLT3-ITD (1.3 nM, Fig. 1b). SKLB-677 also exhibited very good potency against several other selected kinases, including PDGFRα, PDGFRβ, KIT, LOK, VEGFR2, and TIE2. SKLB-677 displayed very weak or no activity at a concentration of 10 μM for other selected kinases, such as CDK2, BTK, DCLK1, ERBB2, MAST1, PAK4, PLK2, ROCK1, and TGFBR1. This result excludes the possibility that SKLB-677 is a non-selective cytotoxic agent. A more comprehensive assessment of the kinase selectivity of SKLB-677 was performed by screening this compound against a KINOMEscan panel of 456 kinase binding assays. The results, shown in Fig. 1c and Supplementary Table S3, indicate that SKLB-677 has a broader kinase interaction pattern. We then calculated the absolute selectivity score (S) of SKLB-677, which result is presented in Supplementary Table S4. For comparison, absolute selectivity scores of several typical FLT3 inhibitors and FDA-approved kinase inhibitors are also given in Supplementary Table S4. The results showed that SKLB-677 (S (100 nM) = ~0.127) had poorer selectivity than the highly specific FLT3 inhibitor AC200 (S (100 nM) = 0.028)^18^, and comparable selectivity with the FDA-approved kinase inhibitors sunitinib (S (100 nM) = 0.19) and dasatinib (S (100 nM) = 0.159), but better selectivity than CEP-701 (S (100 nM) = 0.46)^18^.

### *In vitro* anti-viability activity of SKLB-677 against leukemia and solid tumor cells

The anti-viability activity of SKLB-677 was first tested against various leukemia and solid tumor cell lines using MTT assays. SKLB-677 potently inhibited the viability of FLT3-driven AML cell lines, MV4-11 and Molm-13, with IC_50_ values of 0.079 nM and 0.116 nM, respectively (Table 2 and Fig. 1d). It also exhibited weak inhibitory activity against several other cell lines, including KG-1, HL-60, Jurkat, Ramos, Raji, Karpas-299, SU-DHL-6, PC-9, A549, H358, HepG2, and HeLa cells (Table 1 and Supplementary Fig. S2). Negligible activity was observed against the remaining 16 human cancer cell lines. These results indicate that SKLB-677 is more sensitive to AML cells harboring an FLT3-ITD mutation than to other leukemia and solid tumor cell lines tested.

The anti-viability activity of SKLB-677 against primary AML samples was then tested. For this purpose, we collected six primary AML samples (peripheral blood, PB) from six AML patients, respectively, including three FLT3-ITD positive (sample #1, #2, #3) and three FLT3-ITD negative (sample #4, #5, #6) ones. The patients’ information is listed in Supplementary Table S5. For comparison, three normal PB samples were also collected (sample #7, #8, #9). Peripheral blood mononuclear cells (PBMCs) were isolated from each sample in the following assays. As shown in Fig. 1eFig. 1, samples #1, #2, and #3 were sensitive (IC_50_s:1.09 nM, 1.39 nM, and 0.46 nM, respectively), and samples #4, #5, and #6 were insensitive (IC_50_s > 100 nM) to the treatment of SKLB-677. Very similar results were obtained when the samples were treated with the highly potent and selective FLT3 inhibitor AC220; IC_50_ values were 1.07 nM, 7.02 nM, and 0.88 nM for samples #1, #2, and #3, respectively, and >100 nM for all the other three samples. For the normal PBMCs, SKLB-677 did not exhibit inhibitory effect even at a concentration of 10 μM (Fig. 1f). All of these results demonstrated that SKLB-677 as well as AC220 could significantly kill FLT3-ITD positive primary AML cells, but showed very weak or no activity against FLT3-ITD negative primary AML samples and normal PB samples.

### Comparison between the potency of SKLB-677 and that of other FLT3 inhibitors in clinical development

To compare the potency of SKLB-677 with that of other FLT3 inhibitors in clinical development, we used the same kinase binding assays and cell viability assays described. We included AC220, CEP-701, MLN-518, sorafenib, and sunitinib in this comparison^18^. In the kinase binding assays, all compounds showed high binding affinities for both FLT3 and FLT3-ITD (Table 3). We found that sunitinib was the most potent compound, followed by SKLB-677 and then AC220. In the cell viability assays, all of the compounds tested displayed a high anti-viability potency against FLT3-ITD driven AML cell lines, MV4-11 and Molm-13, and SKLB-677 was the most active one.

### Signaling inhibition in MV4-11 cells and primary AML cells

**Figure 2:**
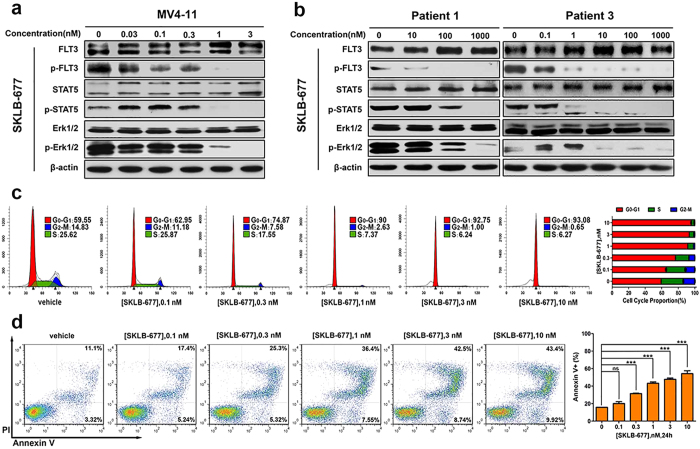
SKLB-677 inhibited FLT3 activation and induced cell cycle arrest and apoptosis. (**a**) MV4-11 cells were treated with various concentrations of SKLB-677 or 0.1% DMSO for 1 h. The phosphorylation levels of FLT3 and downstream signal proteins were assessed using antibodies against total FLT3 (FLT3), phosphorylated Y589/591 FLT3 (p-FLT3), total STAT5 (STAT5), phosphorylated Y694 STAT5 (p-STAT5), total p44/42 MAPK (Erk1/2), and phosphorylated Y202/204 p44/42 MAPK (p-Erk1/2). β-actin was used as a loading control. (**b**) Primary PBMCs isolated from AML patients (FLT3-ITD positive) were treated with various concentrations of SKLB-677 or 0.1% DMSO for 6 h. The phosphorylation levels of FLT3 and downstream signal proteins were detected used the same method as that in panel a. (**c**) MV4-11 cells were treated with various concentrations of SKLB-677 or 0.1% DMSO for 24 h. Cell cycle analysis was performed using propidium iodide staining by flow cytometry. Data shown in the histogram are means ± SD from three independent experiments. (**d**) MV4-11 cells were cultured in the presence of SKLB-677 or 0.1% DMSO, followed by the detection of apoptosis by using Annexin-V and PI co-staining. The early and late apoptotic cells are shown in the right lower and right upper quadrant, respectively. The assays were performed in triplicate, and the data in the histogram represent average percentage of AnnexinV-positive cells ± SD. ns, not statistically significant, ***P < 0.005 vs. vehicle.

Western blot analysis was performed to assess the ability of SKLB-677 to inhibit the activation of FLT3 and downstream signaling proteins in intact MV4-11 cells. After 1 h treatment with increasing concentrations of SKLB-677, MV4-11 cells were harvested and lysed for western blot assays. As shown in Fig. 2a, a dose-dependent reduction in the phosphorylation level of FLT3 was observed following SKLB-677 treatment, with an IC_50_ value of about 0.1 nM. The activation of downstream signaling proteins STAT5 and Erk1/2 was also efficiently inhibited at concentrations of SKLB-677 higher than 1 nM. Similar effects were also observed in primary AML samples (n = 2, Fig. 2b) when treated for 6 h with SKLB-677, although the dose required for the different FLT3-ITD positive patient samples varied substantially, suggesting that other factors influence drug uptake/efflux or activity (Fig. 2b, e.g. patient 1 responded at 1000 nM, patient 3 showed a response at the much lower dose of 10 nM). AC220 also significantly suppressed the phosphorylation of FLT3, STAT5, and Erk1/2 in MV4-11 cells and FLT3-ITD positive AML patient samples (Supplementary Fig. S3).

### Cell cycle and apoptosis assays

PI staining was used to detect the cell cycle status. After incubation with a series of concentrations of SKLB-677 for 24 h, MV4-11 cells exhibited a dose-dependent increase in the percentage of G_0_-G_1_ cells and a dose-dependent decrease in the percentage of G2-M and S phase cells (Fig. 2c). These results indicate cell cycle arrest in G_1_. The induction of apoptosis following treatment of MV4-11 cells was examined by AnnexinV/PI staining assays. As shown in Fig. 2d, SKLB-677 treatment led to a concentration dependent change in the number of apoptotic MV4-11 cells, and a concentration of 3 nM SKLB-677 induced apoptosis of about 50% AML cells. These results indicate that SKLB-677 can induce cell cycle arrest in G_1_ and cause apoptosis at concentrations above the IC_50_ value for MV4-11 cells.

### *In vitro* effects of SKLB-677 on the Wnt/β-catenin signaling

As mentioned above, SKLB-677 was intentionally designed and screened to target both FLT3 and the Wnt/β-catenin pathway. We thus evaluated the *in vitro* effects of SKLB-677 on the Wnt/β-catenin signaling and examined the possible influences of SKLB-677 on leukemia stem-like cells *in vitro*.

**Figure 3:**
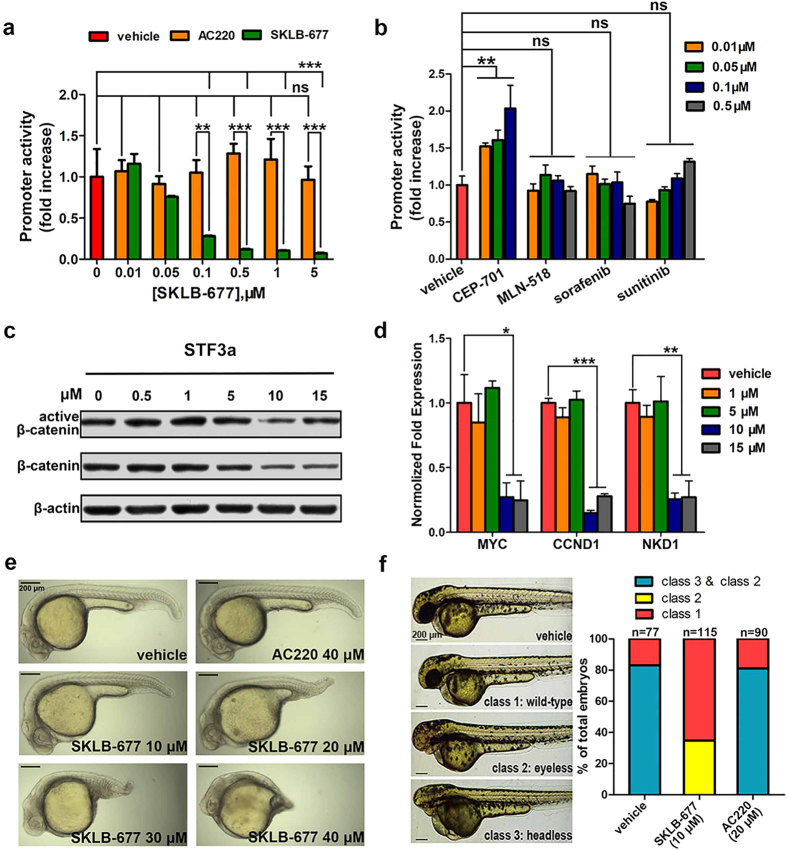
Effects of SKLB-677 on Wnt/β-catenin signaling. (**a**) The inhibitory activity of SKLB-677 against Wnt/β-catenin pathway was determined using STF3a cells treated with various concentrations of SKLB-677 and/or AC220 for 24 h before luciferase activity measurements. All data points are means of triplicates ± SD. ns, not statistically significant, **P < 0.01, ***P < 0.005 among groups. (**b**) The same STF3a cell assays were performed with CEP-701, MLN-518, sorafenib, or sunitinib using the indicated concentrations. ns, not statistically significant, **P < 0.01. (**c**) STF3a cells were incubated with various concentrations of SKLB-677 for 24 h.The cell lysates were detected using the antibodies against S33/37/T41 Non-phospho β-catenin (active β-catenin), and total β-catenin (β-catenin). (**d**) STF3a cells were treated with indicated concentrations of SKLB-677 for 24 h. MYC, CCND1, and NKD1 mRNA expressions were determined through real-time qPCR analysis. All samples were normalized to the level of GAPDH (mean ± SEM from triplicate reactions). *P < 0.05, **P < 0.01, ***P < 0.005. (**e**) Representative images of zebrafish embryos at 24hpf with tail formation after incubation with various conc**e**ntrations of SKLB-677 for 20 h. (**f**) Zebrafish embryos were injected with *wnt8.1* mRNA (30 pg) or vehicle mRNA (phaenotype control). The *wnt8.1* mRNA injected embryos were incubated with 0.1% DMSO, SKLB-677 (10 μM) and AC220 (20 μM) at 4 hpf for 44 h. Representative images of zebrafish embryos at 48 hpf are shown. Results shown in the histogram are expressed as the percentage of embryos in each phenotypic class after the treatment (n > 70). Class 1: wild-type, class 2: posteriorized, resulting in the loss of the eyes, and class 3: more severely posteriorized, as evidenced by head truncation.

The cell line STF3a, which stably expresses both the STF (SuperTopFlash) luciferase reporter promoter and the wnt3a gene was used to determine whether SKLB-677 can block the Wnt/β-catenin signaling. The results, shown in Fig. 3a, indicate that SKLB-677 inhibited the Wnt/β-catenin signaling with an IC_50_ value of <0.1 μM. To determine whether the blockade of Wnt/β-catenin signaling was due to the inhibition of FLT3, the same experiment was performed with STF3a cells and AC220. No obvious attenuation of Wnt/β-catenin signaling was observed (P > 0.05, Fig. 3a). Here we can conclude that the SKLB-677-mediated blockade of Wnt/β-catenin signaling is not due to FLT3 inhibition. We then examined whether the first-generation FLT3 inhibitors could inhibit Wnt/β-catenin signaling. Similar to AC220, the first-generation FLT3 inhibitors tested, including CEP-701, MLN-518, sorafenib, and sunitinib, did not induce obvious attenuation of Wnt/β-catenin signaling (Fig. 3b).

To confirm the Wnt/β-catenin inhibition effect of SKLB-677, we examined the protein levels of total β-catenin and non-phospho-β-catenin (active β-catenin); the non-phospho β-catenin other than the phosphorylated β-catenin enters the cell nucleus and plays the gene regulation function. As shown in Fig. 3c, the protein levels of the total β-catenin and active β-catenin were all slightly decreased following the treatment of SKLB-677 at concentrations of 10 μM or above in STF3a cells. We further examined the expressions of β-catenin target genes MYC, CCND1, and NKD1 by real-time qPCR. The results indicated that MYC, CCND1, and NKD1 transcripts were also reduced in STF3a cells treated with SKLB-677 (Fig. 3d). Finally, the real-time qPCR assays were used again to inspect the influence of SKLB-677 on Wnt/β-catenin signaling in the primary AML cells. The results showed that SKLB-677 treatment also could considerably decrease the expression of β-catenin target genes, indicating inhibition of the Wnt/β-catenin signaling (Supplementary Fig. S4). All of these demonstrated that SKLB-677 could efficiently inhibit the Wnt/β-catenin signaling *in vitro*.

We then performed zebrafish development experiments to further confirm the effect of SKLB-677 on the Wnt/β-catenin signaling. These experiments were based on the observation that blockade of Wnt/β-catenin signaling can effectively inhibit posterior axis formation in zebrafish embryos^28^. SKLB-677 or AC220 was added to the aquarium water at 4hpf. After 24hpf, we observed that SKLB-677 but not AC220 led to embryo dorsalization in a dose-dependent manner (more than 10 embryos in each group, Fig. 3e). The same experiments described above were repeated using the first-generation FLT3 inhibitors. Similar to AC220, the first-generation FLT3 inhibitors tested did not lead to dorsalization, with only one exception that CEP-701 caused severe malformation and development delay (Supplementary Fig. S5). To further confirm the inhibition of Wnt/β-catenin signaling by SKLB-677, zebrafish rescue experiments were conducted. It has been established that the ectopic activation of Wnt/β-catenin signaling can cause the reduction of dorsal mesodermal fates, leading to headless (severe) or eyeless (moderate) zebrafish embryos based on dosage^29^. Therefore, we microinjected *wnt8* ORF 1 (*wnt8.1*) RNA into 1-cell-stage embryos and observed that 82% of the embryos exhibited severely or moderately posteriorized phenotypes (more than 70 zebrafish embryos in each group). When the embryos were treated with 10 μM SKLB-677 at a later stage after the microinjection of *wnt8.1* RNA, 65% of the embryos were unaffected and 35% had only mild or moderate posteriorized phenotypes. This result indicates that SKLB-677 can rescue the eyeless and headless phenotypes caused by the ectopic activation of the Wnt/β-catenin pathway (Fig. 3f). This rescue was not observed when embryos were treated with 20 μM AC220.

### *In vitro* effects of SKLB-677 on leukemia side population (SP) cells and leukemia-initiating cells (LICs)

**Figure 4:**
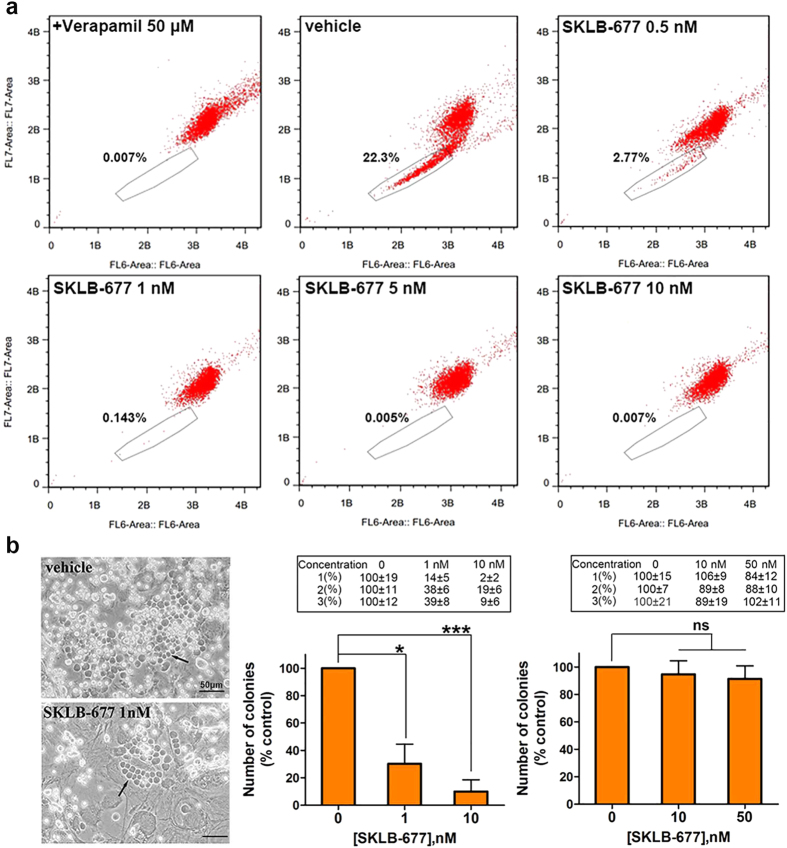
The *in vitro* effects of SKLB-677 on leukemia side population (SP) cells and leukemia-initiating cells (LICs). (**a**) MV4-11 cells were stained with Hoechst 33342 after a treatment of SKLB-677 for 12 h. The ratio of SP cells was 22.3% (**a**s boxed) in the vehicle group, and this ratio was reduced to 0.007% when the cells were co-treated with 50 μM verapamil (positive control). (**b**) The separated PBMCs were incubated on M2-10B4 stromal cells with SKLB-677 (1 nM and 10 nM for primary AML PBMCs, 10 nM and 50 nM for normal PBMCs) and/or 0.1% DMSO and then half medium replaced weekly for six weeks in long-term culture-initiating cells (LTC-IC) assays. Representative CAFCs (cobble stone area forming cells, the clusters of LTC-IC cells) are shown (left, black arrows). The histogram shows the statistics data of three different samples (middle, primary AML PBMCs, n = 3; right, normal PBMCs, n = 3, error bars, ± SEM), and the original data of each sample is also displayed (the table above the histogram, mean ± SD from triplicate assays). ns, not statistically significant, *P < 0.05, ***P < 0.005.

Flow cytometry and Hoechst 33342 dye efflux assays were used to examine the possible influences of SKLB-677 on leukemia SP cells. This method is based on the findings that stem cells and early progenitors are able to pump out Hoechst via the ATP-Binding Cassette (ABC) transporters. This property allows the observation of a cell population (called side population) that has low Hoechst fluorescence in the blue and red regions of the spectrum^30^. In this study, the Hoechst SP cell assays were performed with human AML MV4-11 and KG-1 cells, as well as primary AML PBMCs. Verapamil (50 μM), an ABC transporter inhibitor, was used as a positive control. As shown in Fig. 4a and Supplementary Fig. S6, MV4-11, KG-1, and primary AML PBMCs contained about 22.3%, 3.1%, and 0.5% SP cells, respectively. SKLB-677 treatment obviously reduced the populations of SP cells; 5 nM of SKLB-677 in MV4-11 cells or 5 μM of SKLB-677 in KG-1 cells and primary AML PBMCs could almost eliminate SP cells, and the inhibitory effect was dose-dependent.

In addition to the SP cell assay, we also evaluated the effects of SKLB-677 on the long-term culture-initiating (LTC-IC) cells; the long-term culture of LTC-IC cells can be used as surrogate markers of self-renewal capacity^31^. SKLB-677 treated primary AML PBMCs or normal PBMCs were co-cultured with M2-10B4 stromal cells for 6 weeks, and then the colony formation capacity of LTC-IC cells were determined. In this assay, three primary AML PBMC samples and three normal PBMC samples were included, and each sample was conducted in triplicate. As shown in Fig. 4b, in all the three primary AML PBMC samples, the average LTC-IC colony numbers of three samples were reduced compared with that in the control group after the treatment of SKLB-677 (1 nM SKLB-677: 30%, P < 0.05; 10 nM SKLB-677: 10%, P < 0.005). Conversely, the average LTC-IC colony numbers of three normal PBMC samples were almost not changed after SKLB-677 treatment (P > 0.05).

To examine whether SKLB-677 has influence on normal HSCs and normal blood cells, we performed an experiment using a Balb/c mouse model, in which Balb/c mice (n = 8 in each group) were treated with indicated concentrations of SKLB-677 for 21 days, followed by detections of the proportion of HSCs (immunophenotypically characterized as Lineage^−^Sca-1^+^ c-Kit^+^ (LSK) cells), white blood cell (WBC) and red blood cell (RBC) counts. The results indicated that the HSCs were not inhibited in mice treated with SKLB-677, and the white and red blood counts in these mice were normal during the period of treatment (Supplementary Fig. S7).

### *In vivo* effects of SKLB-677

**Figure 5:**
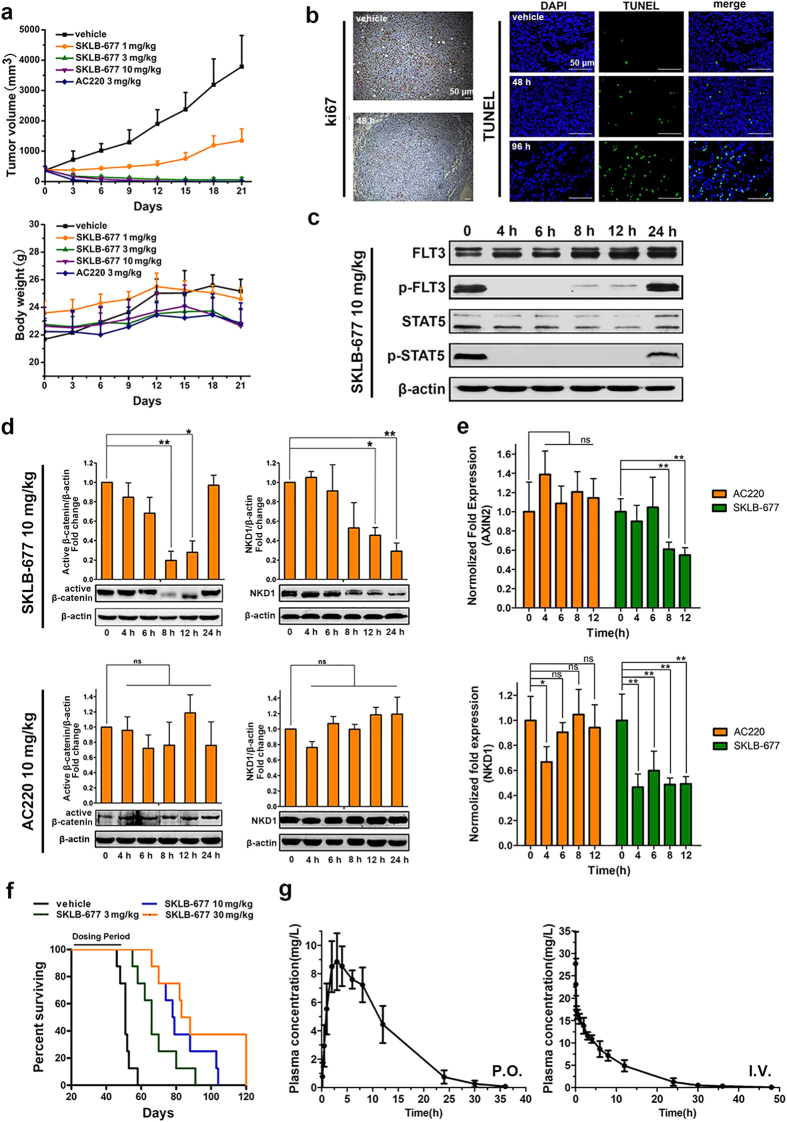
*In vivo* effects of SKLB-677. (**a**) MV4-11 cells (10^7^ cells/mouse) were s.c. inoculated into NOD-SCID mice. The mice were assigned to 5 groups (n = 6 in each group). The tumor volume **a**nd body weight were measured every 3 days during the treatment. The mean values ± SD are shown. (**b**) After the treatment of SKLB-677 for indicated time points, the MV4-11 tumors were collected separately (n = 4 per group). Ki67 and TUNEL were detected with immunohistochemical. Representative images were shown. (**c**) NOD-SCID mice carrying established MV4-11 tumor xenografts were given a single oral dose of SKLB-677 (10 mg/kg). Tumors were harvested at the indicated time points, and the FLT3 signaling pathway was detected using western blot assays. The experiments were performed on three groups of mice. The results of the other two groups are given in Supplementary Data (Supplementary Fig. S8). (**d**) NOD-SCID mice carrying established MV4-11 tumor xenografts were given a single oral dose of SKLB-677 (10 mg/kg) and/or AC220 (10 mg/kg). Tumors were harvested at the indicated time points, and the antibodies against S33/37/T41 Non-phospho β-catenin (active β-catenin) and NKD1 were used to assess the effect on Wnt/β-catenin pathway. The results shown in the histogram are the statistic data of three groups of mice. ns, not statistically significant, *P < 0.05, **P < 0.01. (**e**) The same harvested tumors were detected using real-time qPCR assays to test the expression of AXIN2 and NKD1 (the target genes of Wnt/β-catenin pathway). All results were normalized to the level of GAPDH (mean ± SEM from triplicate groups). ns, not statistically significant, *P < 0.05, **P < 0.01. (**f**) Kaplan-Meier plot of survival. Survival was determined by observation when the animals demonstrated hind-limb paralysis and became moribund. (**g**) Preliminary pharmacokinetic characteristics of SKLB-677. SD rats (n = 6 per group) were orally treated (P.O.) or injected intravenously (I.V.) with 10 mg/kg SKLB-677, followed by the collection of blood from the jugular vein into heparinized tubes at the appropriate times. The plasma concentrations were determined using liquid–liquid extraction followed by high-performance liquid chromatography (HPLC) with tandem mass spectrometric detection.

The *in vivo* anti-leukemia activities of SKLB-677 were evaluated in the FLT3-ITD-dependent MV4-11 tumor xenograft model using AC220 as a positive control. The animals were treated orally once per day for 21 days, and the tumor volumes were measured every 3 days. Treatment with SKLB-677 at 10 mg/kg/d for 3 days or at 3 mg/kg/d for 8 days resulted in complete tumor regression without obvious toxicity (Fig. 5a). SKLB-677 treatment at 1 mg/kg/d led to a tumor inhibition rate of 66%. For the AC220-treated group, a once-daily oral dose at 3 mg/kg/d for 9 days also resulted in complete tumor regression. Obviously, SKLB-677 exhibited a comparable potency with AC220 in the xenograft AML models.

Comparative histopathological analyses of tumor samples from the control and SKLB-677-treated groups were performed to evaluate the anti-tumor mechanism of action for SKLB-677. As shown in Fig. 5b, the SKLB-677-treated tumors contained evidently fewer Ki67-positive cells compared with tumors from the control group, indicating that SKLB-677 inhibited tumor cell proliferation. The percentage of TUNEL-positive cells in the tumors from the SKLB-677-treated group was also increased compared with vehicle-treated tumors, suggesting that SKLB-677 induced apoptosis.

To examine the effects of SKLB-677 on FLT3 and Wnt/β-catenin signaling *in vivo*, the same MV4-11 xenograft model was used and MV4-11 tumors were harvested at specific time points (0 h, 4 h, 6 h, 8 h, 12 h, and 24 h) after the treatment of 10 mg/kg SKLB-677 (3 mice for each time point). Western blot assays and real-time qPCR assays were conducted on the harvested tumors. As shown in Fig. 5c and Supplementary Fig. S8, the phosphorylation of FLT3 and STAT5 were nearly abolished at 4 h posttreatment (the first time point) and the effects were maintained for up to 24 h. SKLB-677 also induced the reduction of active β-catenin and β-catenin target gene NKD1 (Fig. 5d), suggesting inhibition of the Wnt/β-catenin signaling. This result was confirmed by real-time qPCR assays, in which Wnt/β-catenin target genes AXIN2 and NKD1 were found to be down-regulated (Fig. 5e). On the contrary, oral treatment of 10 mg/kg AC220 couldn’t decrease the protein levels of the active β-catenin and NKD1, and the mRNA level of NKD1 and AXIN2 (Fig. 5d,e). All of these demonstrated that SKLB-677 could inhibit both FLT3 and Wnt/β-catenin signaling in the *in vivo* MV4-11 xenograft model.

Finally, the influence of SKLB-677 on survival time was examined using a bone marrow (BM) transplant model of MV4-11. The statistical survival times are shown in Fig. 5f. In the vehicle control group, the mean survival time (MST) was 51 days, with all animals expired by day 58. Compared with the vehicle group, SKLB-677-treated mice demonstrated prolonged survival in a dose-dependent manner with mean survival times (MSTs) of 66, 78.5, and 85.5 days for the 3, 10, and 30 mg/kg/day groups, respectively. Moreover, histopathological analyses of mouse femur BM showed that the number of Ki67-positive cells obviously decreased following SKLB-677 treatment for 6 days (Supplementary Fig. S9), indicating that SKLB-677 could suppress the AML cells in BM.

### Preliminary pharmacokinetic characteristics of SKLB-677

Preliminary pharmacokinetic characteristics of SKLB-677 were assessed on rats with SKLB-677 at a dose of 10 mg/kg P.O. and I.V. administration. The plasma concentration versus time profile is presented in Fig. 5g. The key pharmacokinetic parameters calculated are summarized in Supplementary Table S6. In the P.O. treatment group, SKLB-677 was well absorbed, achieving a maximum plasma level (C_max_) of 12.28 mg/l within 3 h after administration of an oral dose. The area under the plasma drug concentration-time curve (AUC), a measure of total drug exposure, was approximately 175.7 mg/l h. The apparent plasma half-life was 5.4 h. After I.V. treatment at a dose of 10 mg/kg, SKLB-677 displayed a clearance of 0.037l/h/kg with a T_1/2_ of 5.1 h. The apparent volume of distribution (Vd) was 0.271l/kg (<5l/kg), indicating that most of the compound was distributed in body water, which is beneficial to the therapy of leukemia *in vivo*.

## Discussion

SKLB-677 is a new anti-AML agent that has been explicitly optimized to inhibit FLT3 and block Wnt/β-catenin signaling. It exhibited low nanomolar binding affinity for FLT3, and significant anti-viability potency against FLT3-driven AML cells. SKLB-677 also showed potent *in vivo* anti-AML activity in AML animal models. In particular, SKLB-677 but not first-generation and second-generation FLT3 inhibitors in clinical trials has the ability to inhibit Wnt/β-catenin signaling. As expected, SKLB-677 displayed considerable potencies in suppressing leukemia stem-like cells in *in vitro* functional assays. More importantly, it has almost no influence on normal HSCs as well as normal blood cells.

Here it is necessary to mention that, though SKLB-677 can inhibit both FLT3 and the Wnt/β-catenin signaling, the potency of this compound against FLT3 (low nanomolar IC_50_ values) is much stronger compared with that against the Wnt/β-catenin signaling (micromolar IC_50_ values). It is reasonable to deduce that the FLT3 inhibition should be the main contributor of the killing effect of SKLB-677 on AML cells. Despite that a number of studies have shown that Wnt/β-catenin pathway inhibitors could induce apoptosis in cultured AML cells^32^,^33^, we still can not definitely estimate whether the Wnt/β-catenin inhibition by SKLB-677 contributes to the killing effect on AML cells and how much it is. This is because of the discrepancies for the concentrations necessary to efficiently inhibit FLT3 and the Wnt/β-catenin signaling.

One may notice that, though SKLB-677 has been shown to be able to suppress the leukemia stem-like cells in *in vitro* functional assays, the mice in the MV4-11 bone marrow engraftment model still died from leukemia. Possible explanations are given as follows. Firstly, the ability of SKLB-677 to block the Wnt/β-catenin signaling is not sufficiently powerful, and complete inhibition of the Wnt/β-catenin signaling may need a sustained and high concentration of SKLB-677. However, in the MV4-11 bone marrow engraftment model, SKLB-677 was administrated orally once daily, and the mice were just treated by 30 days. Despite that the MST of mice was significantly extended compared with that of untreated mice, the mice still died from leukemia, suggesting that LSCs might not be completely suppressed by the 30 day treatment. Secondly, the regulatory mechanisms of LSCs are quite complicated, and many signal pathways besides the Wnt/β-catenin pathway might be involved. Though several studies by other groups^25^,^34^ and our *in vitro* functional assays in this investigation have clearly demonstrated that the inhibition of Wnt/β-catenin pathway could lead to the suppression of LSCs, it seems difficult to achieve a complete elimination of LSCs. This is in accordance with the results of LTC-IC assays that the colony formation of SKLB-677 treated group was decreased but did not entirely disappear. Finally, it is important to point out that, despite that several *in vitro* functional assays including SP and LTC-IC assays have been performed in this investigation, more rigorous and advanced assays such as secondary transplantation assays using engrafted AML cells are needed in later studies to demonstrate the effects of SKLB-677 on leukemia stem cells.

Finally, we have to acknowledge that in this study just limited AML patient samples were used, and variable response of these samples to the drug was found. It is difficult to define what factors might determine whether a particular FLT3-ITD positive sample would respond to the drug from the limited data. Nevertheless, several factors might be important according to the study by Levis *et al.*^35^. These factors include the AML status of patients (newly diagnosed or relapsed), whether the FLT3-ITD AML cells is truly addicted to FLT3 signaling, and the mutant allelic burden in primary cells. Additionally, though a few of evidence provided here has demonstrated that SKLB-677 can inhibit Wnt/β-catenin signaling, the molecular mechanism is unclear. In fact, an efficient inhibition of the Wnt/β-catenin signaling needs a high concentration (10 μM or above) of SKLB-677. In levels of such high dose of SKLB-677, a number of kinases might be inhibited according to the kinase selectivity profiling data (Table 1 and Supplementary Table S3). Therefore, a possible mechanism could be that SKLB-677 blocks the Wnt/β-catenin signaling through inhibiting some of the kinases; previous studies have demonstrated that many kinases are involved in the regulation of Wnt/β-catenin signaling^36^,^37^,^38^,^39^. Of course, other mechanisms are also possible. For example, SKLB-677 might play its role in suppressing the Wnt/β-catenin signaling through interacting with some components of the Wnt/β-catenin pathway, or regulating the expressions of these genes. Overall, further studies are still needed in the future to uncover the mechanisms underlying the inhibition of Wnt/β-catenin signaling by SKLB-677. Lastly, despite that the targeting of Wnt/β-catenin of SKLB-677 has been demonstrated, the inhibitory activity *in vivo* was transient (the inhibition was obvious after an oral dose of SKLB-677 for 8 h, and back to baseline since then, Fig. 5d). This phenomenon could be mainly due to the *in vivo* pharmacokinetic characters of SKLB-677. Therefore, in order to achieve a sustained inhibitory activity of the Wnt/β-catenin signaling, further optimizations to the pharmacokinetic properties or administration methods might be necessary before clinical studies.

To sum up, SKLB-677 is a new FLT3 inhibitor and has the ability to block the Wnt/β-catenin signaling. This compound exhibited potent anti-AML activity both *in vitro* and *in vivo*. In *in vitro* functional assays, it displayed considerable potency in suppressing leukemia stem-like cells, but had no impact on normal HSCs. Collectively, SKLB-677 could be a promising new lead compound for the treatment of AML. However more mechanistic and pre-clinical studies are still necessary before considering clinical trials.

## Materials and Methods

The human and mouse experiments were performed in accordance with standard guidelines.

### Clinical samples

Methods used in this study were approved by the Ethics Committee of the West China Hospital. Informed consent for the use of human AML and normal peripheral blood (PB) samples for the present study was obtained from all patients according to the Declaration of Helsinki. Primary AML samples evaluated in this study were taken from six AML patients (sample #1, #2, #3: FLT3-ITD positive; sample #4, #5, #6: FLT3-ITD negative), and normal PB samples were taken from three normal volunteers (sample #7, #8, #9). PBMCs were isolated using density gradient centrifugation with Ficoll-Hypaque (DAKEWE) within 24 h of collection.

### Cell lines

All cell lines used were obtained from American Type Culture Collection (ATCC), with the exception of STF3a, which was kindly provided by Professor David M. Virshup (Program in Cancer and Stem Cell Biology, Duke-NUS Graduate Medical School)^40^. These cell lines were cultured in culture medium supplemented with 10% FBS (Caoyuanlvye), 100 U/ml penicillin, and 100 U/ml streptomycin. All cell lines were maintained at 37 °C in a CO_2_ incubator with 5% CO_2_.

### Compounds

CEP-701, MLN518, sorafenib, sunitinib, and AC220 were purchased from Nanjing Chemlin Chemical Industry Co. Ltd (Nanjing, Jiangsu). SKLB-677 and compound **1** were synthesized at the State Key Laboratory of Biotherapy, Sichuan University (Chengdu, Sichuan). All compounds used in this research were of analytical purity and culture grade.

### Biochemical kinase binding assays

*In vitro* KINOMEscan kinase binding assays (Ambit Biosciences) were employed to determine the binding affinity (Kd) of compounds^41^,^42^.

### Cell viability assay

Effects of compounds on the cell viability of tumor cell lines were examined using a cell viability reagent, MTT (Sigma). Briefly, tumor cells were seeded in a 96-well plate in the absence or presence of tested compounds for 72 h. MTT (5 mg/ml in sodium chloride) was added for the last 2 h of incubation, and the absorbances at 570 nm were measured after the dissolve of DMSO. The IC_50_ values were calculated by GraphPad Prism 5.01 software (GraphPad Software, USA).

Effects of compounds on the PBMC viability were examined using CellTiterGlo (Promega) assays. PBMCs were seeded in 96-well plates at a density of 5 × 10^4^ cells per well prior to the addition of different concentrations of SKLB-677 or AC220. After 72 h of incubation, the cells were analyzed for viability by the addition of CellTiterGlo reagent to the assay plates. The signal from the viable cells was assessed according to the manufacturer’s protocol.

### Cell cycle and apoptosis assays

Flow cytometry was used for cell cycle and apoptosis assays. MV4-11 cells were treated with SKLB-677 for 24 h, and then the cells were harvested and washed with PBS. Cell Cycle and Apoptosis Analysis Kit (Beyotime) was used for cell cycle analysis and the Annexin V-FITC & PI Cell Apoptosis Analysis Kit (KeyGENE BioTECH) was used for apoptosis assays. The data was analyzed using FLOWJO software (V7.6.5).

### Western blot analysis

Western blot analysis was performed as previously described^43^. Whole protein lysates of tumor samples were collected by the abrading of tumors in liquid nitrogen. All the samples were separated by sodium dodecyl sulfate-polyacrylamide gel electrophoresis (SDS-PAGE), and probed overnight at 4 °C with the following primary antibodies: FLT3 (1:500, Santa Cruz Biotechnology, sc-480), Phospho-FLT3(Tyr589/591) (1:1000, Cell Signaling, #3464), STAT5 (1:1000, Cell Signaling, #9363), Phospho-STAT5(Tyr694) (1:1000, Cell Signaling, #9314), p44/42 MAPK (1:1000, Cell Signaling, #4695), Phospho-p44/42 MAPK(Thr 202/Tyr204) (1:1000, Cell Signaling, #4370), Non-phospho β-catenin (Ser33/37/Thr41) (1:1000, Cell Signaling, #8814), β-catenin (1:500, Santa Cruz Biotechnology, sc-59737), NKD1 (1:10000, abcam, ab133650) and β-actin (1:1000, ZSGB-BIO, TA-09). Quantitative analysis of the protein level was performed with NIH image analysis software, ImageJ (normalized relative to the vehicle control).

### Luciferase assay

STF3a cells (8 × 10^3^) were seeded overnight in 96-well plates (triplicate wells per treatment). Cells were treated with 0.1% DMSO or tested compounds for 24 h with Luciferase assay system (Promega) performed according to the manufacturer’s instructions. No obvious toxicity was observed at the test point in each well. The reporter activity was expressed as relative values compared with vehicle control.

### Real-time qPCR analysis

Total RNA of cell samples and tumor samples was extracted using RNAsimple total RNA kit (TIANGEN) according to the manufacturer’s instructions. cDNA was generated using the iScript^TM^cDNA Synthesis Kit (Bio-Rad), and the RT-PCR reactions were performed using SsoAdvanced SYBR Green Supermix (Bio-Rad). The reaction consisted of 40 cycles of denaturation at 95 °C for 5 s and annealing/extension for 30s at 60 °C. The primer sequences are listed in Supplementary Table S7.

### Transgenic zebrafish

Embryos were obtained according to the standard procedures from WT (AB*) strain fish. Capped *wnt8.1* mRNAs were synthesized using the appropriate mMessage mMachine Kit (Ambion), following the manufacturer’s protocol. Synthesized mRNAs of *wnt8.1* (30 pg) were injected into the yolk of 1-cell embryos. The assigned concentrations of compounds were added at 4hpf (hours post-fertilization). At 48hpf, photos of the embryo morphology were taken under a microscope (LEICA DM 2500) by a digital camera (LEICA DFC425 C). Image acquisition software: LAS V3.7.

### Isolation of SP cells

For the SP cell analysis, MV4-11 cells were plated in 6-well plates and treated with SKLB-677 for 12 h. Then, the harvested cells were stained with the fluorescent dye Hoechst 33342 (Sigma-Aldrich) at a final concentration of 5 μg/ml in a 37 °C water bath for 60 min. After 60 min, the cells were centrifuged at 4 °C and resuspended in ice-cold HBSS (containing 2 μg/ml PI (Sigma)). SP cells were detected using a Beckman MofloXDP flow cytometer.

### Long-term culture-initiating cell (LTC-IC) assay

PBMCs from AML patients (n = 3) and normal people (n = 3) were plated on M2-10B4 feeder cell layers (irradiated with 8000cGy) established in 6-well plates in HLTM medium (Myelocult H5100, STEMCELL) freshly supplemented with 10^−6^ M hydrocortisone (STEMCELL). Cultures were maintained with 0.1% DMSO or SKLB-677 for 5 weeks with weekly half-media changes, before seeding harvested cells into Colony-forming unit assays (cells were seeded in ultra-low attachment 6-well plates (Corning) in triplicate with the complete MethoCult Media (STEMCELL) for 14 days). The assay for each sample was performed in triplicate. The colonies were counted under a microscope, and an obvious and visible cell cluster was considered a colony. The representative images were taken on an Axiovert 200 microscope with an AxiocamMRm camera with 0.30 aperture. Image acquisition software: AxioVisionRell 4.8.

### *In vivo* models

#### Flank and engraftment model

Female 5- to 6-week-old NOD-SCID mice (Beijing HFK Bioscience) were housed in a sterile environment and fed a standard diet *ad libitum*. MV4-11 cells were harvested and resuspended at a concentration of 1 × 10^8^ cells/ml in serum-free medium. A 100- μl aliquot of the cell suspension was injected subcutaneously into the hind flank of each mouse to establish the xenograft model. The mice bearing tumors were randomized into 5 groups (6 mice/group) when the tumors grew to a size of ~400 mm^3^. The mice were then dosed orally with SKLB-677 (1, 3, or 10 mg/kg/d), AC220 (3 mg/kg) or vehicle. The compounds were dissolved in 12.5% (v/v) castor oil and 12.5% ethanol in 75% sterile water. Tumor growth was measured every 3 days to monitor the therapeutic effect of SKLB-677. The tumor volume was calculated using the following formula: volume = a × b^2^/2 (a, long diameter; b, short diameter).

#### Bone marrow engraftment model

NOD-SCID mice were pretreated with cyclophosphamide by intraperitoneal injection of 150 mg/kg/d for 2 days. Following a 24-hour rest period, the mice were intravenously injected with 8 × 10^6^ MV4-11 cells per mouse via the tail vein. From the day 21 after inoculation, SKLB-677 or vehicle was administered orally once a day for 30 days. Survival was determined by observation when the animals demonstrated hind-limb paralysis and became moribund.

#### Immunohistochemical analysis

The tumor tissues were dissected from the sacrificed NOD-SCID mice after treatment with SKLB-677 for the indicated time. Paraffin-embedded tumors were subjected to immunostaining with Ki67 (Thermo Fisher), transferase-mediated dUTP nick-end labeling (TUNEL, Thermo Fisher). The representative images were taken on a LEICA DM 2500 microscope with a LEICA DFC425 C camera. Image acquisition software: LAS V3.7.

#### The pharmacokinetic assessments

SD rats (6 per group) were orally treated or injected intravenously with 10 mg/kg SKLB-677, followed by the collection of blood from the jugular vein into heparinized tubes at the appropriate times. The plasma was isolated by centrifugation. The plasma concentrations of SKLB-677 were determined using liquid–liquid extraction followed by high-performance liquid chromatography (HPLC) with tandem mass spectrometric detection. Noncompartmental pharmacokinetic parameters (AUC, T_max_, C_max_, and t_1⁄2_) were obtained from the blood concentration time profiles using DAS software.

### Statistical analysis

Data were expressed as mean ± SD or mean ± SEM. Unless specifically mentioned, all experiments were performed in triplicate. For data analyses, two-tailed Student’s *t*-tests were performed using GraphPad Prism 5.01. In all comparisons, P < 0.05 was considered statistically significant.

## Additional Information

**How to cite this article**: Ma, S. *et al.* SKLB-677, an FLT3 and Wnt/β-catenin signaling inhibitor, displays potent activity in models of FLT3-driven AML. *Sci. Rep.* **5**, 15646; doi: 10.1038/srep15646 (2015).

## Supplementary Material

Supplementary Information

## Figures and Tables

**Table 1:** Binding affinities (Kds) of SKLB-677 with FLT3, various FLT3 mutants, and a set of selected kinases.

Kinase	SKLB-677 (Kd, nM)	Kinase	SKLB-677 (Kd, nM)
FLT3-wt	0.74	MET	220
FLT3-D835H	0.74	FGFR1	600
FLT3-D835Y	1.3	CDK2	>10000
FLT3-ITD	1.3	BTK	>10000
PDGFRα	12	DCLK1	>10000
PDGFRβ	2.7	ERBB2	>10000
KIT	3.1	MAST1	>10000
LOK	5.1	PAK4	>10000
VEGFR2	11	PLK2	>10000
TIE2	99	ROCK1	>10000
LCK	130	TGFBR1	>10000

**Table 2:** Anti-viability activity of SKLB-677 against various tumor cells.

Cancer type	Cell line	Characteristic	IC_50_, μM
Leukemia(AML)	MV4-11	FLT3-ITD	0.000079
Leukemia(AML)	Molm-13	FLT3-ITD	0.000116
Leukemia(AML)	THP-1	MLL-AF9 fusion	>10
Leukemia(AML)	KG-1	FGF-FGFR loop	2
Leukemia(APL)	HL-60	NA	8.9
Leukemia(ALL)	Jurkat	PTEN deficient	4.3
Myeloma	U266	Produce IL-6	>10
Lymphoma	Ramos	NA	8.4
Lymphoma	Raji	MYC-IGH fusion	7.9
Lymphoma	Karpas-299	NPM1-ALK fusion	7
Lymphoma	SU-DHL-6	EZH2 mutantion	7.783
NSCLC	PC-9	EGFR mutation	8.026
NSCLC	H1975	EGFR T790M	>10
NSCLC	H441	KRAS mutation	>10
NSCLC	A549	KRAS G12S	6.4
NSCLC	H358	KRAS mutation	8
Pancreatic	Miapaca-2	KRAS mutation	>10
Pancreatic	PANC-1	KRAS mutation	>10
Liver	HepG2	Aurora B mutation	6.704
Liver	SMMC7721	NA	>10
Liver	BEL7402	NA	>10
Liver	PLC/PRF/5	NA	>10
Cervix carcinoma	HeLa	NA	9.448
Colon	SW620	KRAS mutation	>10
Melanoma (*Mus*)	B16	NA	>10
Melanoma	WM2664	NA	>10
Melanoma	A2058	NA	>10
Melanoma	C32	NA	>10
Breast	MCF-7	PI3K mutation	>10
Breast	MDA-MB-231	KRAS G13D	>10

NA, not applicable.

**Table 3:** Potencies of FLT3 inhibitors in biochemical and cellular assays.

Compound	Biochemical assay (Kd, nM)		Cell viability (IC_50_, nM)	
FLT3	FLT3-ITD	MV4-11	Molm-13	
SKLB-677	0.74	1.3	0.079	0.116
AC220	1.3	8.8	0.29	0.750
CEP-701	8.5	1.5	1.92	1.410
MLN-518	3	9.1	39.42	109.8
Sorafenib	13	79	3.099	1.055
Sunitinib	0.41	0.99	2.171	1.669
