# Supplementary material for: SKLB-677, an FLT3 and Wnt/β-catenin signaling inhibitor, displays potent activity in models of FLT3-driven AML
Source: Sci Rep. 2015 Oct 26;5:15646. doi: 10.1038/srep15646 (PMC4620497; doi:10.1038/srep15646)
Supplement: Supplementary Information [file srep15646-s1.pdf]

SKLB-677, an FLT3 and Wnt/β-catenin signaling inhibitor, displays potent activity in models of FLT3-driven AML

Shuang Ma[1,+], Ling-Ling Yang[1,+], Ting Niu[1,2,+], Chuan Cheng[1], Lei Zhong[1], Ming-Wu Zheng[1], Yu Xiong[1], Lin-Li Li[1], Rong Xiang[3], Li-Juan Chen[1], Qiao Zhou[1], Yu-Quan Wei[1], and Sheng-Yong Yang[1,]*

[1]State Key Laboratory of Biotherapy and Cancer Center, West China Hospital, Sichuan University/Collaborative Innovation Center of Biotherapy, Chengdu, 610041, China

[2]Department of Hematology & Research Laboratory of Hematology, West China Hospital, Sichuan University, Chengdu, 610041, China

[3]Department of Clinical Medicine, School of Medicine, Nankai University, Tianjin, 300071, China

+these authors contributed equally to this work.

*corresponding author: Dr. Sheng-Yong Yang, Address: State Key Laboratory of Biotherapy and Cancer Center, West China Hospital, Sichuan University/Collaborative Innovation Center of Biotherapy, Chengdu, Sichuan, 610041, P. R. China; Phone: 86-28-85164063; Fax: 86-28-85164060; Email: yangsy@scu.edu.cn

Supplementary Material and Methods

Chemistry

Unless otherwise noted, all starting materials, reagents, and solvents were purchased from commercial vendors and used as supplied without further purification. Reactions were monitored by thin layer chromatography (TLC) on Merck silica gel 60 F-254 thin layer plates. ^{1}H NMR and ^{13}C NMR spectra were recorded on a Bruker AV-400 spectrometer at 400 MHz and 100 MHz, respectively. Chemical shifts (δ) are expressed in parts per million (ppm) relative to tetramethylsilane (TMS) as an internal standard. The following abbreviations are used to designate multiplicities: s, singlet; d, doublet; t, triplet; q, quartet; m, multiplet; dd, double–doublet; brs, broad signal. Low-resolution and high-resolution mass spectral (MS) data were determined on an Agilent 1100 Series LCMS with UV detection at 254nm in low-resonance electrospray mode (ESI). All final compounds were purified to >95% purity, as determined by high-performance liquid chromatography (HPLC). HPLC analysis was performed on a Waters 2695 HPLC system equipped with a Kromasil C18 column (4.6mm × 250mm, 5um).

Isolation of SP cells in cultured KG-1 cells and primary AML cells

KG-1 cells and primary AML cells were plated in 6-well plates and incubated at 37°C 5% CO_2 overnight, followed by treatment with SKLB-677 for 48h. Then, the cells were harvested, and stained with Hoechst 33342 (Beyotime) at a final concentration of 5μg/ml without washing. The cells were mixed by gentle inversion and incubated in a 37°C water bath for 90min. After 90min, the cells were centrifuged at 4°C and resuspended in ice-cold HBSS (containing 2μg/ml PI (Sigma)). SP cells

were detected using BD FACSAriaIII flow cytometer.

Isolation of murine Bone Marrow (BM) cells.

Balb/c mice were grouped to 4 groups (4 female mice and 4 male mice per group) and orally treated with 0, 1, 3, and 10mg/kg/d SKLB-677 for 21 days. For the isolation of total BM, mice were sacrificed at the last time point, and the tibias and femurs were removed. BM cells were washed out with RPMI-1640 medium, filtered through a 100-μm cell strainer and centrifuged for 5min. Subsequently, the cell pellet was resuspended in 1ml ACK Lysis Buffer (Beyotime) and incubated for 5min at room temperature to allow lysis of red blood cells. Then, cells were washed in PBS and HSCs were analyzed.

Detection of HSCs by flow cytometry

BM cells were incubated with antibodies of Lineage-APC (CD3e, CD11b, CD45R/B220, Ly-76, Ly-6G, Ly-6C), Sca-1-PE and c-Kit-FITC in 4°C for 30min. All the antibodies were purchased from Becton Dickinson. After the incubation, the cells were then washed twice with PBS and analyzed on a BD LSR II flow cytometer. FACS data were analyzed with FlowJo software.

Chemistry of SKLB-677

Supplementary Scheme S1 [a]

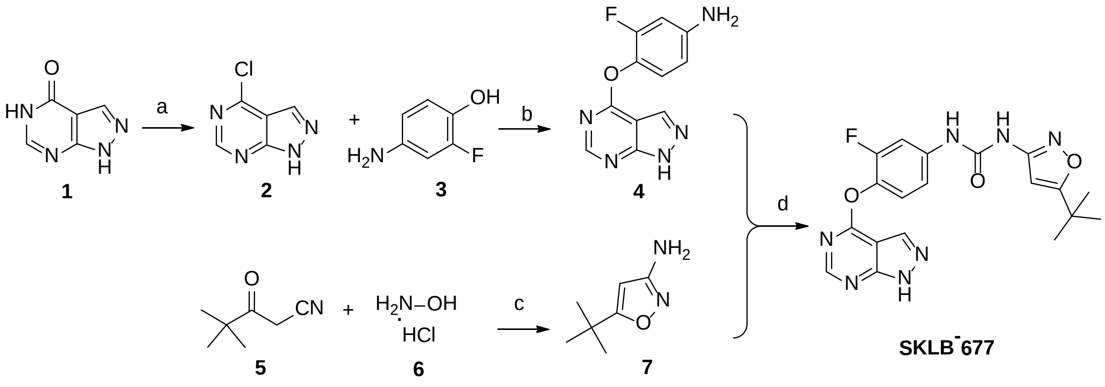

[a] Reagents and conditions: (a) phosphorus oxychloride, ethyldiisopropylamine, toluene, reflux, 56%; (b) 4-amino-2-fluorophenol, sodium hydroxide, H_2O, tetrahydrofuran, 25°C -60°C, 41%; (c) NaOH, H_2O, HCl, 25°C -55°C, 38%; (d) Acetonitrile, toluene, 110°C, 16h, 89%.

Synthetic routes for the target compound SKLB-677 are depicted in Supplementary Scheme S1. Commercially available compound 1*H*-pyrazolo[3,4-*d*]pyrimidin- 4(5*H*)-one (**1**) reacted with phosphorus oxychloride to produce the intermediate 4-chloro-1*H*-pyrazolo[3,4-*d*]pyrimidine (**2**). Sodium hydroxide (0.20g, 5.0mmol) was added to a solution of 4-amino-2-fluorophenol (**3**, 0.64g, 5.0mmol) in H_2O (10ml). The mixture was stirred at room temperature for 0.5h. Then, 4-chloro-1*H*-pyrazolo[3,4-*d*]pyrimidine (**2**, 0.77g, 5.0mmol) in tetrahydrofuran (20ml) was slowly added and the reaction mixture was heated to 60°C for 1.5h. The solvent was partially evaporated on a rotary evaporator. The crude mixture was extracted with ethyl acetate (2×120ml) and water. The combined organic layers were dried over $MgSO_4$ and concentrated. The obtained residue was purified by column chromatography (eluent gradient dichloromethane: methanol, 80:1) without final recrystallization to give 4-(1H-pyrazolo[3,4-d]pyrimidin-4-yloxy)-3-fluoroaniline (**4**, 0.502g, 41%) as a brown solid.

The intermediate compound 5-Tert-butylisoxazol-3-amine (**7**) was prepared by initial attack of the hydroxylamine nitrogen on the cyano group of the 4,4-dimethyl-3-oxopentanenitrile (**5**) at pH 7-8 at 55°C, followed by ring closure under acidic conditions (pH 3-4) at 55°C (Yield: 38%).

A solution of 5-tert-butylisoxazol-3-amine (**7**, 0.70g, 5.0mmol) dissolved in tetrahydrofuran (30ml) was slowly dripped into a stirred solution of triphosgene (1.49g, 5.0mmol) in tetrahydrofuran (5ml) using a constant pressure dropping funnel. Triethylamine (1.5ml, 10.5mmol) was then added slowly to the reaction mixture after the aniline was completely added. After evaporation of the solvent, the residue was dissolved in acetonitrile (20ml) and toluene (40ml), and compound **4** (1.10g, 4.5mmol) was added. Next, the reaction mixture was stirred at 110°C overnight, and the solvent was removed *in vacuo*. The residue obtained was purified by column chromatography (eluent gradient petroleum ether: ethyl acetate, 3:1) and recrystallized from ethyl acetate and petroleum ether to provide the final product SKLB-677. Yield: 89%, 99% HPLC purity.^{1}H NMR (400 MHz, DMSO-d_6): δ 14.27 (s, 1H), 9.63 (s, 1H), 9.13 (s, 1H), 8.51 (s, 1H), 8.29 (s, 1H), 7.67 (d, *J*=10.4 Hz, 1H), 7.41 (t, *J*=8.8 Hz, 1H), 7.23 (d, *J*=8.8 Hz, 1H), 6.50 (s, 1H) 1.30 (s, 9H) ppm; ^{13}C NMR (100 MHz, DMSO-d_6): δ 180.3, 162.1, 158.2, 156.7, 154.8, 152.3, 151.3, 138.2, 133.3, 131.7, 124.3, 114.8, 106.8, 100.9, 92.5, 32.4, 28.3 ppm; LCMS m/z: 412.2 [M + H]; 99% HPLC purity.

Supplementary Figures and Legands

Supplementary Figure S1.

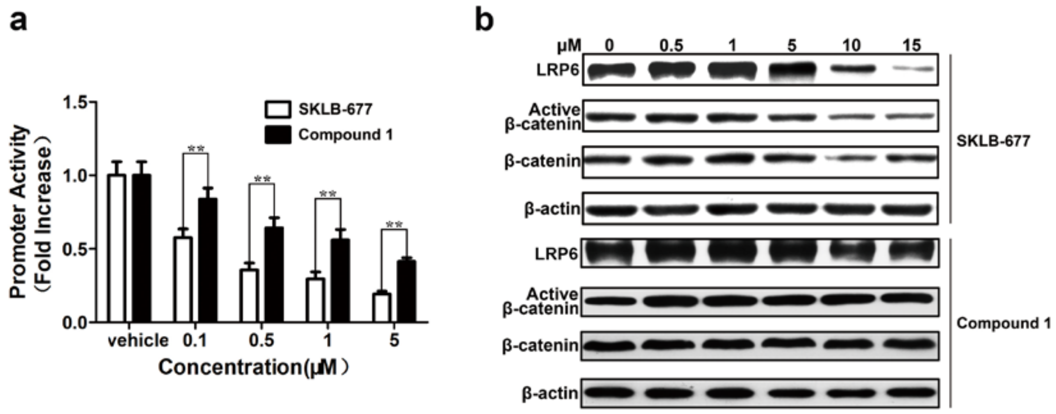

Supplementary Figure S1. A comparison of the inhibitory activity against Wnt/β-catenin pathway between SKLB-677 and compound **1**. a, STF3a cells were treated with SKLB-677 and/or compound **1** for 24h, followed by the detection of luciferase activity as mentioned in the text. Experiments were performed in triplicate, and the error bars indicate the SD values. ** $P<0.01$. b, STF3a cells were treated with SKLB-677 and/or compound **1** for 24h, and the protein level of Wnt/β-catenin associated proteins was detected using specific antibodies.

Supplementary Figure S2

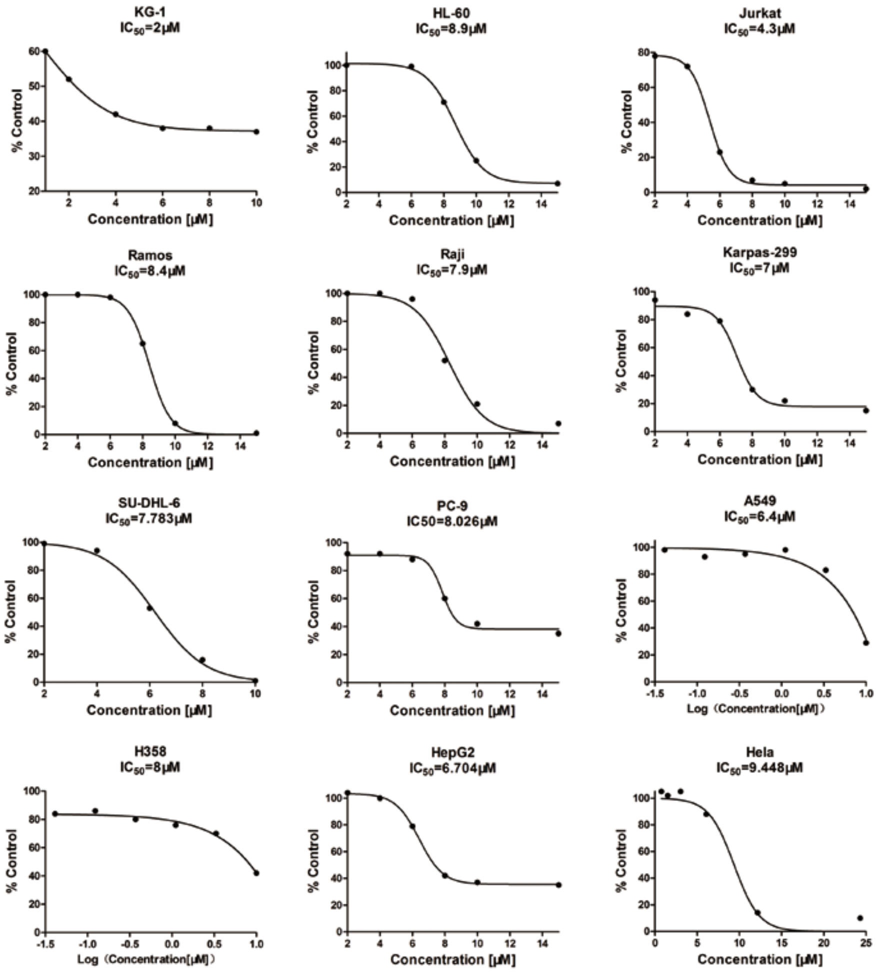

Supplementary Figure S2. The dose-response curves for various tumor cell lines treated with SKLB-677. Cells were treated with indicated concentration of SKLB-677 for 72h, followed by the conduction of MTT assays.

Supplementary Figure S3.

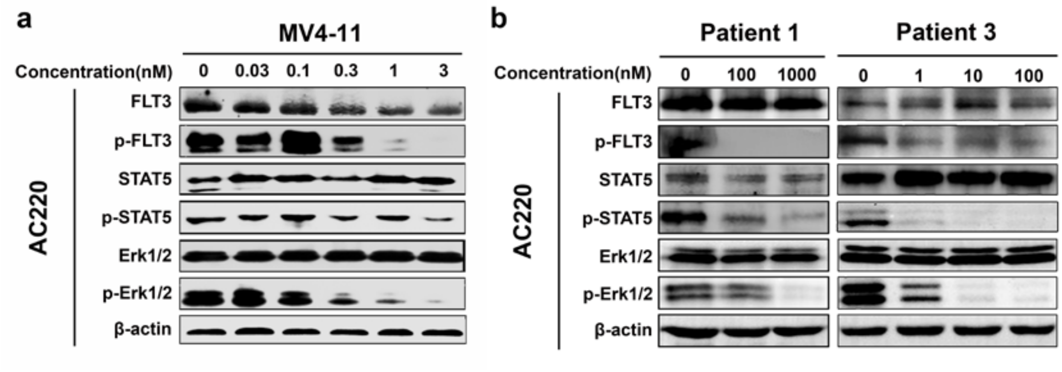

Supplementary Figure S3. a, MV4-11 cells was treated with various concentration of AC220 or 0.1%DMSO for 1h. The phosphorylated and un-phosphorylated FLT3 and its downstream signal proteins were assessed. β-actin was used as a loading control. b, Primary cells isolated from AML patients (sample #1, #3: FLT3-ITD positive) were treated with various concentrations of AC220 or 0.1%DMSO for 6h. The phosphorylated and un-phosphorylated FLT3 and its downstream signal proteins were detected using western blotting assays.

Supplementary Figure S4

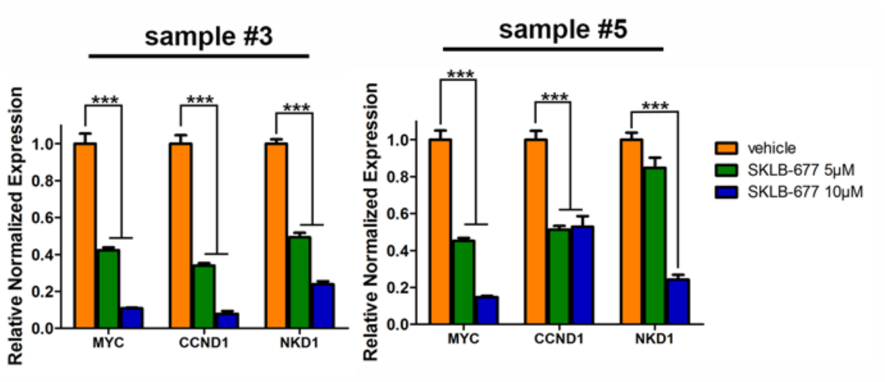

Supplementary Figure S4. The real-time qPCR assays in primary AML cells. Primary AML PBMCs were treated with indicated concentrations of SKLB-677 for 6h. The mRNA expression levels of β-catenin target genes MYC, CCND1 and NKD1 were determined through real-time qPCR analysis. All samples were normalized to the level of GAPDH (mean ±SEM from triplicate reactions). *** P<0.005.

Supplementary Figure S5.

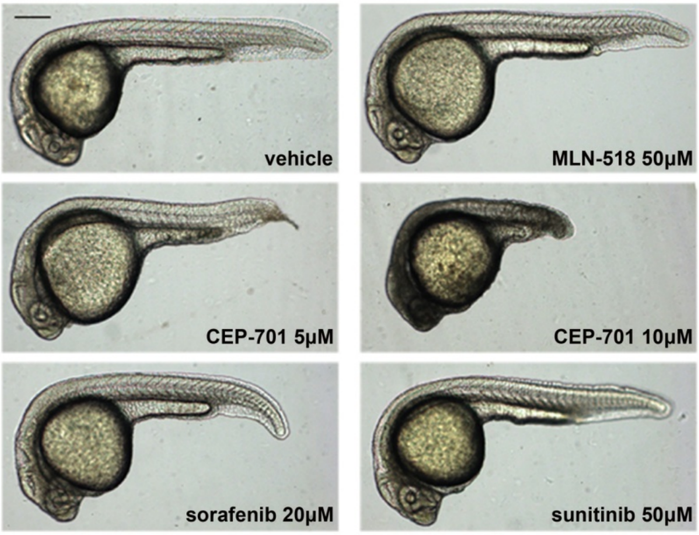

Supplementary Figure S5. Zebrafish development experiments were used to examine the effect of the first-generation FLT3 inhibitors including CEP-701, MLN-518, sorafenib, and sunitinib on the Wnt/β-catenin signaling. CEP-701, MLN-518, sorafenib, or sunitinib was added to the fish water at 4hpf (more than 10 zebrafish embryos in each group). The representative images were taken after 24hpf. CEP-701 caused severe malformation and development delay. Scale bar, 200μm.

Supplementary Figure S6

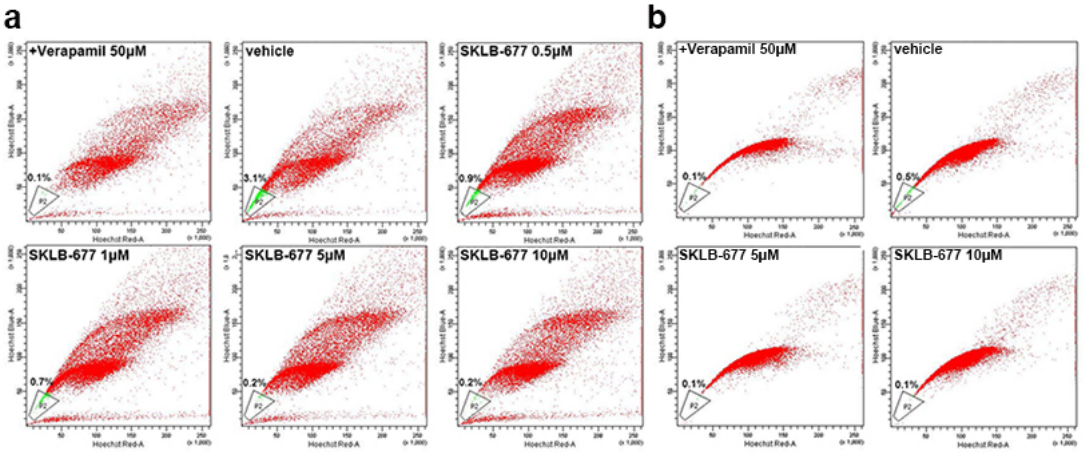

Supplementary Figure S6. Side population (SP) cell assays in cultured KG-1 cells and primary AML cells. a, KG-1 cells were incubated with SKLB-677 for 48h and stained with Hoechst 33342. The ratio of SP cells was 3.1% (as boxed) in the vehicle group, and this ratio was reduced when the cells were co-treated with 50μM verapamil. b, Primary AML PBMCs were isolated from peripheral blood using the standard protocol and incubated with SKLB-677 for 48h, following by the staining with Hoechst 33342. 50μM verapamil was used as a positive control.

Supplementary Figure S7.

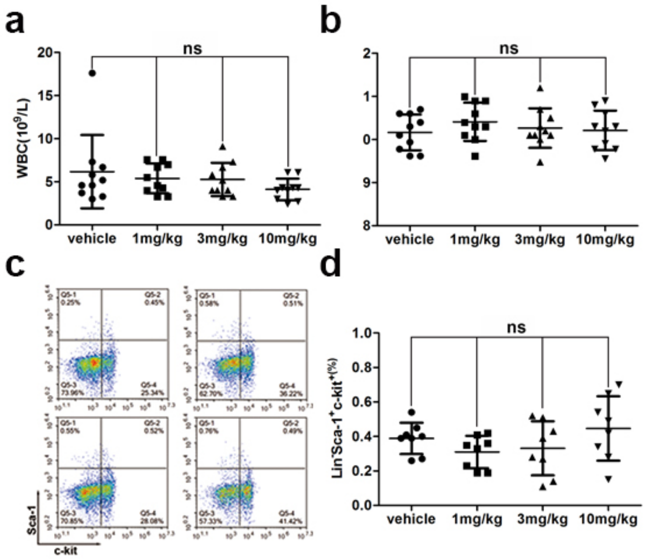

Supplementary Figure S7. The influence of SKLB-677 on mouse normal HSC and blood cells (8 mice in each group). a, b, The mice were orally treated with indicated concentrations of SKLB-677 for 21d, and then WBC and RBC were counted using a blood analyzer instrument. ns, not statistically significant. c, The mice were orally treated with indicated doses of SKLB-677 for 21d, and the blood marrow cells were collected for the detection of HSC (immunophenotypically characterized as Lineage-Sca-1+c-Kit+, LSK) population using corresponding antibodies. d, The statistical data of HSC numbers. ns, not statistically significant.

Supplementary Figure S8

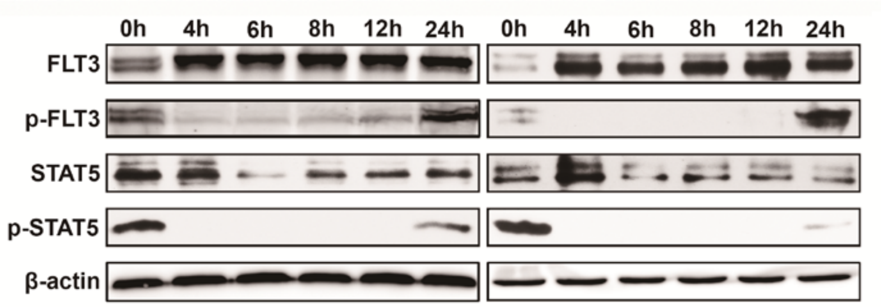

Supplementary Figure S8. NOD-SCID mice carrying established MV4-11 tumor xenografts were given a single oral dose of SKLB-677 (10mg/kg) or vehicle control. Tumors were harvested at the indicated time points, and the FLT3 signaling pathway was detected using western blot assays. Each lane is for one mouse. The experiments were performed on three mouse groups. Results for one group are shown in Fig. 5c, and those for the other two groups are shown here.

Supplementary Figure S9.

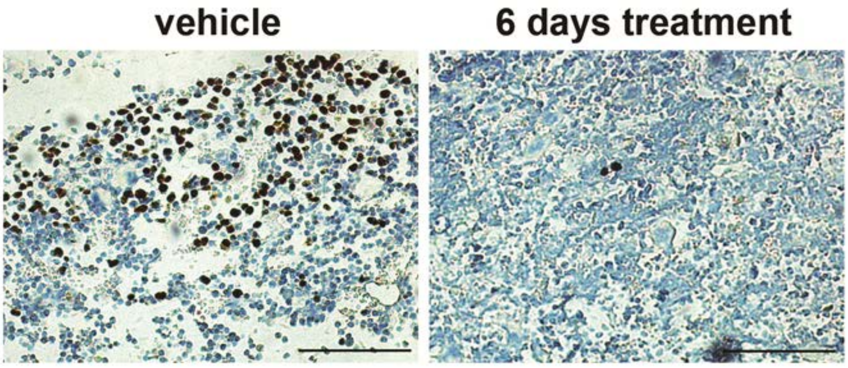

Supplementary Figure S9. NOD-SCID mice were intravenously injected with $8×10^6$ MV4-11 cells per mouse via the tail vein. Oral administration of SKLB-677 at 10 mg/kg/d was initiated on day 21 after the inoculation. Mouse (3 mice per group) thigh femurs were collected for immunohistochemical Ki67 analysis after 6 days of SKLB-677 treatment. The data proved that SKLB-677 significantly induced the regression of bone marrow tumor. Scale bar, 100μm.

Supplementary Tables

A comparison between SKLB-677 and compound 1.

SKLB-677 and compound **1** are analogues, and SKLB-677 was derived from compound **1**. They have very similar spectrum of activity in enzymatic and cellular levels. Both of them have the ability to inhibit Wnt/β-catenin signaling. However, in terms of the potency, SKLB-677 is much more potent than compound **1**.

Supplementary Table S1. Kinase inhibition profiles for SKLB-677 and compound **1**.

**Kinase**	**SKLB-677 (Kd, μM)**	**Compound 1 (IC_{50}, μM)**
FLT3	0.00074	0.039
VEGFR2	0.011	0.012
PDGFRα	0.012	0.223
PDGFRβ	0.0027	0.408
KIT	0.0031	0.507
ERBB2	>10	>10
ERBB4	>10	>10
CDK2	>10	>10
CHEK1	>10	>10
CAMK4	>10	>10
DLK	>10	>10
DMPK	>10	>10
ERN1	>10	>10
IGF1R	>10	>10
PAK1	>10	>10
PAK2	>10	>10
PAK4	>10	>10
ERK	>10	>10
LCK	0.13	>10

Supplementary Table S2. Cell viability inhibition profiles for SKLB-677 and Compound **1**.

**Tumor Type**	**Cell Line**	**SKLB-677 (IC_{50}, μM)**	**Compound 1 (IC_{50}, μM)**
Leukemia, AML	MV4-11	0.000079	0.004
Leukemia, ALL	Jurkat	4.3	8.72
Leukemia, APL	HL60	8.9	>10
Cervical Cancer	Hela	9.448	>10
Breast Cancer	MCF-7	>10	>10
Liver Cancer	BEL7402	>10	>10
Liver Cancer	SMMC7721	>10	>10
Lung Cancer	A549	6.4	>10
Lymphoma	Raji	7.9	>10
Lymphoma	Karpas-299	7	>10
Multiple myeloma	U266	>10	>10

Supplementary Table S3. Matrix of compound screen for SKLB-677 obtained through service provided by DiscoveRX. The compound was screened at a concentration of 10μM. Results are given in %control.

KINOMEscan Gene Symbol	Entrez Gene Symbol	Percent Control(%)	Compound Concentration (μM)
AAK1	AAK1	72	10
ABL1(E255K)-phosphorylated	ABL1	11	10
ABL1(F317I)-nonphosphorylated	ABL1	2.2	10
ABL1(F317I)-phosphorylated	ABL1	43	10
ABL1(F317L)-nonphosphorylated	ABL1	10	10
ABL1(F317L)-phosphorylated	ABL1	13	10
ABL1(H396P)-nonphosphorylated	ABL1	5.8	10
ABL1(H396P)-phosphorylated	ABL1	11	10
ABL1(M351T)-phosphorylated	ABL1	5.6	10
ABL1(Q252H)-nonphosphorylated	ABL1	5.2	10
ABL1(Q252H)-phosphorylated	ABL1	21	10
ABL1(T315I)-nonphosphorylated	ABL1	3	10
ABL1(T315I)-phosphorylated	ABL1	3.3	10
ABL1(Y253F)-phosphorylated	ABL1	10	10
ABL1-nonphosphorylated	ABL1	1.2	10
ABL1-phosphorylated	ABL1	3.2	10
ABL2	ABL2	13	10
ACVR1	ACVR1	95	10
ACVR1B	ACVR1B	100	10
ACVR2A	ACVR2A	100	10
ACVR2B	ACVR2B	81	10
ACVRL1	ACVRL1	91	10
ADCK3	CABC1	100	10
ADCK4	ADCK4	100	10
AKT1	AKT1	100	10
AKT2	AKT2	100	10
AKT3	AKT3	100	10
ALK	ALK	4.8	10
ALK(C1156Y)	ALK	17	10
ALK(L1196M)	ALK	71	10
AMPK-alpha1	PRKAA1	3	10
AMPK-alpha2	PRKAA2	7.8	10
ANKK1	ANKK1	57	10

ARK5	NUAK1	47	10
ASK1	MAP3K5	57	10
ASK2	MAP3K6	32	10
AURKA	AURKA	35	10
AURKB	AURKB	2.6	10
AURKC	AURKC	1.4	10
AXL	AXL	3.8	10
BIKE	BMP2K	89	10
BLK	BLK	0.95	10
BMPR1A	BMPR1A	90	10
BMPR1B	BMPR1B	88	10
BMPR2	BMPR2	14	10
BMX	BMX	73	10
BRAF	BRAF	2.7	10
BRAF(V600E)	BRAF	0.05	10
BRK	PTK6	23	10
BRSK1	BRSK1	85	10
BRSK2	BRSK2	76	10
BTK	BTK	100	10
BUB1	BUB1	66	10
CAMK1	CAMK1	21	10
CAMK1D	CAMK1D	35	10
CAMK1G	CAMK1G	48	10
CAMK2A	CAMK2A	73	10
CAMK2B	CAMK2B	77	10
CAMK2D	CAMK2D	100	10
CAMK2G	CAMK2G	66	10
CAMK4	CAMK4	85	10
CAMKK1	CAMKK1	4.4	10
CAMKK2	CAMKK2	30	10
CASK	CASK	59	10
CDC2L1	CDK11B	66	10
CDC2L2	CDC2L2	53	10
CDC2L5	CDK13	0.6	10
CDK11	CDK19	0.1	10
CDK2	CDK2	1.6	10
CDK3	CDK3	0.2	10
CDK4-cyclinD1	CDK4	29	10
CDK4-cyclinD3	CDK4	4.8	10
CDK5	CDK5	4.2	10
CDK7	CDK7	3.1	10
CDK8	CDK8	3	10
CDK9	CDK9	5.6	10

CDKL1	CDKL1	3.3	10
CDKL2	CDKL2	1.7	10
CDKL3	CDKL3	0.75	10
CDKL5	CDKL5	5	10
CHEK1	CHEK1	100	10
CHEK2	CHEK2	32	10
CIT	CIT	16	10
CLK1	CLK1	5.8	10
CLK2	CLK2	6.4	10
CLK3	CLK3	52	10
CLK4	CLK4	9.6	10
CSF1R	CSF1R	0.3	10
CSF1R-autoinhibited	CSF1R	0.1	10
CSK	CSK	76	10
CSNK1A1	CSNK1A1	100	10
CSNK1A1L	CSNK1A1L	68	10
CSNK1D	CSNK1D	44	10
CSNK1E	CSNK1E	31	10
CSNK1G1	CSNK1G1	86	10
CSNK1G2	CSNK1G2	26	10
CSNK1G3	CSNK1G3	70	10
CSNK2A1	CSNK2A1	100	10
CSNK2A2	CSNK2A2	71	10
CTK	MATK	37	10
DAPK1	DAPK1	74	10
DAPK2	DAPK2	83	10
DAPK3	DAPK3	71	10
DCAMKL1	DCLK1	100	10
DCAMKL2	DCLK2	100	10
DCAMKL3	DCLK3	81	10
DDR1	DDR1	3.6	10
DDR2	DDR2	61	10
DLK	MAP3K12	86	10
DMPK	DMPK	100	10
DMPK2	CDC42BPG	50	10
DRAK1	STK17A	100	10
DRAK2	STK17B	78	10
DYRK1A	DYRK1A	80	10
DYRK1B	DYRK1B	41	10
DYRK2	DYRK2	34	10
EGFR	EGFR	29	10
EGFR(E746-A750del)	EGFR	37	10
EGFR(G719C)	EGFR	37	10

EGFR(G719S)	EGFR	47	10
EGFR(L747-E749del, A750P)	EGFR	19	10
EGFR(L747-S752del, P753S)	EGFR	38	10
EGFR(L747-T751del,Sins)	EGFR	61	10
EGFR(L858R)	EGFR	29	10
EGFR(L858R,T790M)	EGFR	75	10
EGFR(L861Q)	EGFR	46	10
EGFR(S752-I759del)	EGFR	38	10
EGFR(T790M)	EGFR	74	10
EIF2AK1	EIF2AK1	96	10
EPHA1	EPHA1	9.2	10
EPHA2	EPHA2	1.2	10
EPHA3	EPHA3	24	10
EPHA4	EPHA4	4.8	10
EPHA5	EPHA5	0.7	10
EPHA6	EPHA6	0	10
EPHA7	EPHA7	0	10
EPHA8	EPHA8	0.1	10
EPHB1	EPHB1	3.2	10
EPHB2	EPHB2	5.2	10
EPHB3	EPHB3	0.8	10
EPHB4	EPHB4	1	10
EPHB6	EPHB6	2	10
ERBB2	ERBB2	100	10
ERBB3	ERBB3	98	10
ERBB4	ERBB4	81	10
ERK1	MAPK3	76	10
ERK2	MAPK1	100	10
ERK3	MAPK6	79	10
ERK4	MAPK4	98	10
ERK5	MAPK7	70	10
ERK8	MAPK15	0.8	10
ERN1	ERN1	100	10
FAK	PTK2	84	10
FER	FER	24	10
FES	FES	7.2	10
FGFR1	FGFR1	1.4	10
FGFR2	FGFR2	2	10
FGFR3	FGFR3	12	10
FGFR3(G697C)	FGFR3	22	10
FGFR4	FGFR4	4.8	10
FGR	FGR	9.4	10
FLT1	FLT1	0.05	10

FLT3	FLT3	2	10
FLT3(D835H)	FLT3	0	10
FLT3(D835Y)	FLT3	3.2	10
FLT3(ITD)	FLT3	0.05	10
FLT3(K663Q)	FLT3	1.8	10
FLT3(N841I)	FLT3	0	10
FLT3(R834Q)	FLT3	2.8	10
FLT3-autoinhibited	FLT3	1.2	10
FLT4	FLT4	0	10
FRK	FRK	0	10
FYN	FYN	15	10
GAK	GAK	100	10
GCN2(Kin.Dom.2,S808G)	EIF2AK4	20	10
GRK1	GRK1	97	10
GRK4	GRK4	45	10
GRK7	GRK7	87	10
GSK3A	GSK3A	30	10
GSK3B	GSK3B	96	10
HASPIN	GSG2	42	10
HCK	HCK	0.5	10
HIPK1	HIPK1	68	10
HIPK2	HIPK2	4.4	10
HIPK3	HIPK3	1.2	10
HIPK4	HIPK4	0.45	10
HPK1	MAP4K1	13	10
HUNK	HUNK	80	10
ICK	ICK	42	10
IGF1R	IGF1R	70	10
IKK-alpha	CHUK	75	10
IKK-beta	IKBKB	64	10
IKK-epsilon	IKBKE	55	10
INSR	INSR	87	10
INSRR	INSRR	42	10
IRAK1	IRAK1	2	10
IRAK3	IRAK3	52	10
IRAK4	IRAK4	100	10
ITK	ITK	36	10
JAK1(JH1domain-catalytic)	JAK1	12	10
JAK1(JH2domain-pseudokinase)	JAK1	74	10
JAK2(JH1domain-catalytic)	JAK2	31	10
JAK3(JH1domain-catalytic)	JAK3	14	10
JNK1	MAPK8	52	10
JNK2	MAPK9	2	10

JNK3	MAPK10	18	10
KIT	KIT	0	10
KIT(A829P)	KIT	15	10
KIT(D816H)	KIT	8.4	10
KIT(D816V)	KIT	0.2	10
KIT(L576P)	KIT	0	10
KIT(V559D)	KIT	0	10
KIT(V559D,T670I)	KIT	0.1	10
KIT(V559D,V654A)	KIT	1.8	10
KIT-autoinhibited	KIT	1.1	10
LATS1	LATS1	47	10
LATS2	LATS2	26	10
LCK	LCK	0.3	10
LIMK1	LIMK1	25	10
LIMK2	LIMK2	7	10
LKB1	STK11	96	10
LOK	STK10	0	10
LRRK2	LRRK2	52	10
LRRK2(G2019S)	LRRK2	78	10
LTK	LTK	1.2	10
LYN	LYN	2	10
LZK	MAP3K13	93	10
MAK	MAK	21	10
MAP3K1	MAP3K1	36	10
MAP3K15	MAP3K15	100	10
MAP3K2	MAP3K2	100	10
MAP3K3	MAP3K3	100	10
MAP3K4	MAP3K4	69	10
MAP4K2	MAP4K2	28	10
MAP4K3	MAP4K3	3.2	10
MAP4K4	MAP4K4	0	10
MAP4K5	MAP4K5	2	10
MAPKAPK2	MAPKAPK2	100	10
MAPKAPK5	MAPKAPK5	97	10
MARK1	MARK1	78	10
MARK2	MARK2	98	10
MARK3	MARK3	68	10
MARK4	MARK4	70	10
MAST1	MAST1	100	10
MEK1	MAP2K1	7.2	10
MEK2	MAP2K2	5.9	10
MEK3	MAP2K3	63	10
MEK4	MAP2K4	39	10

MEK5	MAP2K5	13	10
MEK6	MAP2K6	62	10
MELK	MELK	56	10
MERTK	MERTK	0	10
MET	MET	0.05	10
MET(M1250T)	MET	2.2	10
MET(Y1235D)	MET	5.4	10
MINK	MINK1	2.3	10
MKK7	MAP2K7	77	10
MKNK1	MKNK1	0.1	10
MKNK2	MKNK2	1	10
MLCK	MYLK3	58	10
MLK1	MAP3K9	42	10
MLK2	MAP3K10	99	10
MLK3	MAP3K11	24	10
MRCKA	CDC42BPA	100	10
MRCKB	CDC42BPB	90	10
MST1	STK4	28	10
MST1R	MST1R	42	10
MST2	STK3	87	10
MST3	STK24	46	10
MST4	MST4	24	10
MTOR	MTOR	100	10
MUSK	MUSK	0	10
MYLK	MYLK	40	10
MYLK2	MYLK2	72	10
MYLK4	MYLK4	78	10
MYO3A	MYO3A	11	10
MYO3B	MYO3B	17	10
NDR1	STK38	58	10
NDR2	STK38L	56	10
NEK1	NEK1	64	10
NEK10	NEK10	93	10
NEK11	NEK11	96	10
NEK2	NEK2	100	10
NEK3	NEK3	41	10
NEK4	NEK4	38	10
NEK5	NEK5	9.8	10
NEK6	NEK6	99	10
NEK7	NEK7	48	10
NEK9	NEK9	54	10
NIK	MAP3K14	90	10
NIM1	MGC42105	33	10

NLK	NLK	1.2	10
OSR1	OXSR1	36	10
p38-alpha	MAPK14	0	10
p38-beta	MAPK11	0.2	10
p38-delta	MAPK13	10	10
p38-gamma	MAPK12	3.7	10
PAK1	PAK1	68	10
PAK2	PAK2	79	10
PAK3	PAK3	6.4	10
PAK4	PAK4	100	10
PAK6	PAK6	90	10
PAK7	PAK7	100	10
PCTK1	CDK16	0.2	10
PCTK2	CDK17	0	10
PCTK3	CDK18	0.05	10
PDGFRA	PDGFRA	0.25	10
PDGFRB	PDGFRB	0	10
PDPK1	PDPK1	100	10
PFCDPK1(P.falciparum)	CDPK1	26	10
PFPK5(P.falciparum)	MAL13P1.279	100	10
PFTAIRE2	CDK15	0.85	10
PFTK1	CDK14	0.05	10
PHKG1	PHKG1	85	10
PHKG2	PHKG2	100	10
PIK3C2B	PIK3C2B	100	10
PIK3C2G	PIK3C2G	100	10
PIK3CA	PIK3CA	100	10
PIK3CA(C420R)	PIK3CA	96	10
PIK3CA(E542K)	PIK3CA	100	10
PIK3CA(E545A)	PIK3CA	86	10
PIK3CA(E545K)	PIK3CA	100	10
PIK3CA(H1047L)	PIK3CA	84	10
PIK3CA(H1047Y)	PIK3CA	100	10
PIK3CA(I800L)	PIK3CA	87	10
PIK3CA(M1043I)	PIK3CA	100	10
PIK3CA(Q546K)	PIK3CA	100	10
PIK3CB	PIK3CB	100	10
PIK3CD	PIK3CD	98	10
PIK3CG	PIK3CG	100	10
PIK4CB	PI4KB	52	10
PIM1	PIM1	26	10
PIM2	PIM2	37	10
PIM3	PIM3	40	10

PIP5K1A	PIP5K1A	22	10
PIP5K1C	PIP5K1C	51	10
PIP5K2B	PIP4K2B	98	10
PIP5K2C	PIP4K2C	100	10
PKAC-alpha	PRKACA	31	10
PKAC-beta	PRKACB	88	10
PKMYT1	PKMYT1	100	10
PKN1	PKN1	82	10
PKN2	PKN2	100	10
PKNB(M.tuberculosis)	pknB	100	10
PLK1	PLK1	35	10
PLK2	PLK2	100	10
PLK3	PLK3	100	10
PLK4	PLK4	68	10
PRKCD	PRKCD	61	10
PRKCE	PRKCE	90	10
PRKCH	PRKCH	100	10
PRKCI	PRKCI	79	10
PRKCQ	PRKCQ	50	10
PRKD1	PRKD1	98	10
PRKD2	PRKD2	28	10
PRKD3	PRKD3	100	10
PRKG1	PRKG1	86	10
PRKG2	PRKG2	100	10
PRKR	EIF2AK2	57	10
PRKX	PRKX	44	10
PRP4	PRPF4B	73	10
PYK2	PTK2B	64	10
QSK	KIAA0999	91	10
RAF1	RAF1	3.5	10
RET	RET	0.05	10
RET(M918T)	RET	0	10
RET(V804L)	RET	0	10
RET(V804M)	RET	1.9	10
RIOK1	RIOK1	89	10
RIOK2	RIOK2	88	10
RIOK3	RIOK3	100	10
RIPK1	RIPK1	31	10
RIPK2	RIPK2	0.75	10
RIPK4	RIPK4	0.85	10
RIPK5	DSTYK	98	10
ROCK1	ROCK1	100	10
ROCK2	ROCK2	66	10

ROS1	ROS1	0.1	10
RPS6KA4(Kin.Dom.1-N-terminal)	RPS6KA4	29	10
RPS6KA4(Kin.Dom.2-C-terminal)	RPS6KA4	78	10
RPS6KA5(Kin.Dom.1-N-terminal)	RPS6KA5	66	10
RPS6KA5(Kin.Dom.2-C-terminal)	RPS6KA5	100	10
RSK1(Kin.Dom.1-N-terminal)	RPS6KA1	100	10
RSK1(Kin.Dom.2-C-terminal)	RPS6KA1	94	10
RSK2(Kin.Dom.1-N-terminal)	RPS6KA3	35	10
RSK2(Kin.Dom.2-C-terminal)	RPS6KA3	100	10
RSK3(Kin.Dom.1-N-terminal)	RPS6KA2	41	10
RSK3(Kin.Dom.2-C-terminal)	RPS6KA2	19	10
RSK4(Kin.Dom.1-N-terminal)	RPS6KA6	64	10
RSK4(Kin.Dom.2-C-terminal)	RPS6KA6	72	10
S6K1	RPS6KB1	54	10
SBK1	SBK1	80	10
SGK	SGK1	29	10
SgK110	SgK110	100	10
SGK2	SGK2	78	10
SGK3	SGK3	46	10
SIK	SIK1	97	10
SIK2	SIK2	69	10
SLK	SLK	0.2	10
SNARK	NUAK2	66	10
SNRK	SNRK	100	10
SRC	SRC	9.2	10
SRMS	SRMS	57	10
SRPK1	SRPK1	96	10
SRPK2	SRPK2	100	10
SRPK3	SRPK3	71	10
STK16	STK16	36	10
STK33	STK33	0.05	10
STK35	STK35	0.95	10
STK36	STK36	97	10
STK39	STK39	100	10
SYK	SYK	25	10
TAK1	MAP3K7	100	10
TAOK1	TAOK1	3.2	10
TAOK2	TAOK2	0.35	10
TAOK3	TAOK3	0.55	10
TBK1	TBK1	70	10
TEC	TEC	100	10
TESK1	TESK1	82	10
TGFBR1	TGFBR1	100	10

TGFBR2	TGFBR2	100	10
TIE1	TIE1	8.7	10
TIE2	TEK	0.2	10
TLK1	TLK1	75	10
TLK2	TLK2	64	10
TNIK	TNIK	5.2	10
TNK1	TNK1	2.2	10
TNK2	TNK2	37	10
TNNI3K	TNNI3K	0	10
TRKA	NTRK1	7.8	10
TRKB	NTRK2	0.05	10
TRKC	NTRK3	0.05	10
TRPM6	TRPM6	89	10
TSSK1B	TSSK1B	100	10
TTK	TTK	17	10
TXK	TXK	88	10
TYK2(JH1domain-catalytic)	TYK2	44	10
TYK2(JH2domain-pseudokinase)	TYK2	100	10
TYRO3	TYRO3	0.35	10
ULK1	ULK1	70	10
ULK2	ULK2	97	10
ULK3	ULK3	51	10
VEGFR2	KDR	0.1	10
VRK2	VRK2	100	10
WEE1	WEE1	64	10
WEE2	WEE2	78	10
WNK1	WNK1	100	10
WNK3	WNK3	100	10
YANK1	STK32A	88	10
YANK2	STK32B	50	10
YANK3	STK32C	97	10
YES	YES1	27	10
YSK1	STK25	35	10
YSK4	YSK4	0.15	10
ZAK	ZAK	1.5	10
ZAP70	ZAP70	100	10

Supplementary Table S4. Selectivity scores for kinase inhibitors. The selectivity scores (S= number of target hits/number of kinases screened) were calculated as previously described (see Karaman MW, et al. A quantitative analysis of kinase inhibitor selectivity. Nature biotechnology 2008; 26: 127-32) based on the data for distinct kinases excluding mutant variants. The value of S (100nM) of SKLB-677 was derived according to the data provided by DiscoveRX.

Inhibitor	S(3μM)	S(100nM)
SKLB-677	ND	~0.127
AC220	0.067	0.028
CEP-701	0.81	0.46
MLN-518	0.056	0.014
Sorafenib	0.18	0.045
Sunitinib	0.58	0.19
Dasatinib	0.283	0.159
Erlotinib	0.152	0.014
Gefitinib	0.072	0.007
Imatinib	0.066	0.031
Lapatinib	0.010	0.010

ND, not determined.

SupplementaryTable S5. The characteristics of AML patients. Selected genes were detected.

Patient	Age(y)	Sex	AML Status	Cytogenetic Status	Gene Mutation
#1	32	M	Diagnosis, refractory	Normal	FLT3-ITD mutation, NPM1 mutation, $HOX11^{+}$
#2	15	M	Diagnosis, refractory	+8	FLT3-ITD mutation, WT1 mutation
#3	31	F	Newly diagnosed	Normal	FLT3-ITD mutation
#4	44	M	Newly diagnosed	Normal	DNMT3A mutation, PHF2 mutation
#5	57	M	Newly diagnosed	Normal	$BCR\text{-}ABL^{+}$
#6	22	M	Diagnosis, refractory	Normal	TIE2 mutation, ASXL1 mutation, $EVI1^{+}$

Supplementary Table S6. The pharmacokinetic characteristics of SKLB-677. $AUC_{(0\text{-}36h)}$: Mean area under the plasma concentration-time curve during 36h and standard deviation. $t_{1/2}$: Mean half-life associated with the terminal slope and standard deviation. C_{max}: Mean peak plasma concentration and standard deviation.

T_{max}: Mean time to reach maximum plasma concentration and standard deviation. CL: Mean body clearance and standard deviation. V_{ss}: Mean volume of distribution at steady state and standard deviation. The statistical analysis of data was shown as mean values and SD values (n=6).

**Parameter**	**P.O.**		**I.V.**	
	**Mean**	**SD**	**Mean**	**SD**
$AUC_{(0\text{-}36h)}$ (mg/L×h)	175.654	22.903	272.344	31.304
$t_{1/2}$ (h)	5.433	1.318	5.091	0.471
T_{max} (h)	3	0.816	0.033	0
CL (L/h/kg)	0.058	0.007	0.037	0.004
V_{ss} (L/kg)	0.446	0.108	0.271	0.046
C_{max} (mg/L)	12.283	0.762	58.213	5.244

Supplementary Table S7. Primers used in the real time qPCR assays.

Genes	Forward primers (5’-3’)	Reverse primers (5’-3’)
NKD1	GTCAACCACTCCCCAACATC	AATGGTGGTAGCAGCCAGAC
AXIN2	GGGCCACTTTAAAGAGCAG	CCTTCATACATCGGGAGCA
MYC	CGTCTCCACACATCAGCACAA	TCTTGGCAGCAGGATAGTCCTT
CCND1	CCGTCCATGCGGAAGATC	ATGGCCAGCGGGAAGAC
GAPDH	TGGAAGGACTCATGACCACA	TTCAGCTCAGGGATGACCTT
